# Classic Maya response to multiyear seasonal droughts in Northwest Yucatán, Mexico

**DOI:** 10.1126/sciadv.adw7661

**Published:** 2025-08-13

**Authors:** Daniel H. James, Stacy A. Carolin, Sebastian F. M. Breitenbach, Julie A. Hoggarth, Fernanda Lases-Hernández, Erin A. Endsley, Jason H. Curtis, Christina D. Gallup, Susan Milbrath, John Nicolson, James Rolfe, Ola Kwiecien, Christopher J. Ottley, Alexander A. Iveson, James U. L. Baldini, Mark Brenner, Gideon M. Henderson, David A. Hodell

**Affiliations:** ^1^ Godwin Laboratory for Palaeoclimate Research, Department of Earth Sciences, University of Cambridge, Cambridge, UK.; ^2^ Department of Earth Sciences, University of Oxford, Oxford, UK.; ^3^ Department of Geography and Environmental Sciences, Northumbria University, Newcastle upon Tyne, UK.; ^4^ Department of Anthropology, Baylor University, Waco, TX, USA.; ^5^ Facultad de Química, Universidad Nacional Autónoma de México, Mérida, Yucatán, Mexico.; ^6^ Swenson College of Science and Engineering, University of Minnesota Duluth, Duluth, MN, USA.; ^7^ Department of Geological Sciences and Land Use and Environmental Change Institute, University of Florida, Gainesville, FL, USA.; ^8^ Florida Museum of Natural History, University of Florida, Gainesville, FL, USA.; ^9^ Department of Earth Sciences, University of Durham, Durham, UK.

## Abstract

Protracted droughts may have contributed to sociopolitical upheaval and depopulation of cultural centers in the Maya Lowlands during the Terminal Classic Period (~800 to 1000 CE). Regional proxy climate records suggest multiple prolonged drought episodes during the Terminal Classic. The relationship between drought and response of individual sites, however, remains unclear because of large chronological uncertainties and poor temporal resolution of existing local paleoclimate inferences. We present a subannual rainfall record from northwest Yucatán, Mexico, derived from an annually laminated stalagmite spanning 871 to 1021 CE, with ±6-year age uncertainty. Interpretation of the stalagmite oxygen isotope record is supported by modern rain and drip water monitoring. Precisely dated droughts enable detailed analyses of timing and dynamics of regional human-climate interactions. Despite uncertainties in archaeological chronologies, these results suggest political activity at major northern Maya sites, including Chichén Itzá and Uxmal, declined at different times relative to droughts, implying differential cultural responses to climate stress.

## Teaser

Stalagmite record reveals 1- to 13-year droughts in NW Yucatán from 871 to 1021 CE, a time of profound Maya cultural change.

## INTRODUCTION

During the Maya Terminal Classic Period (~800 to 1000 CE), large urban centers in the Southern Lowlands of the Yucatán Peninsula experienced widespread sociopolitical upheaval, site abandonment, and depopulation (*1*–*3*). The center of political power shifted northward, and construction of dated monuments (stelae) faltered, ceasing entirely by 998 CE (*4*, *5*). Debate continues as to the causes of these sociopolitical changes, including the role played by climate change (*6*–*11*). Paleoclimate records from lake sediments and speleothems suggest that frequent droughts of 1- to 10-year duration occurred during the Terminal Classic (*12*–*17*). Meteorological droughts (reductions in annual precipitation amount) (*18*) were previously invoked to explain these cultural changes (*7*), but singular causes are inconsistent with the considerable spatial and temporal complexity of both archaeological and paleoclimate evidence (*9*, *11*, *14*, *19*, *20*). For example, the major decline in Maya population in the Central and Southern Lowlands during the 9th and 10th centuries coincided with the rise of some polities in the drier north (*21*). This seemingly contradictory pattern is often cited as evidence against drought having been the sole cause of Maya cultural transformation in the Terminal Classic. Sharpening the regional picture requires highly resolved, accurately dated records of both paleoclimate and archaeological change that can be compared directly at the local scale and placed in a broader sociopolitical context in the region (*22*).

**Fig. 1.**
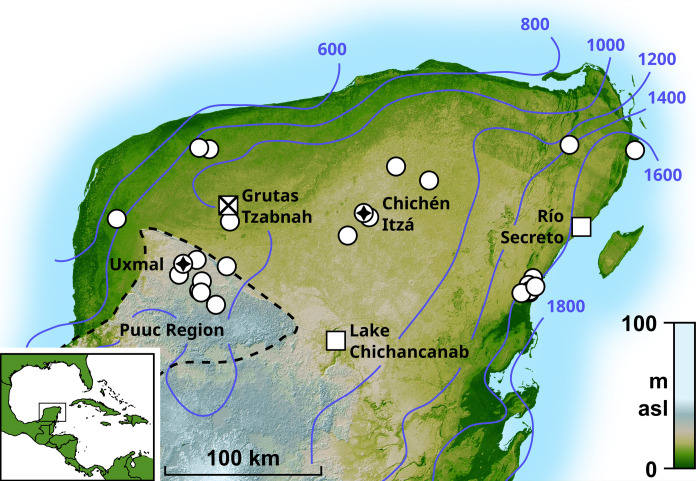
Map of the Maya Lowlands. Numbered contours are modeled mean annual total rainfall 1979–2022, in millimeters per year (ECMWF ERA5, European Centre for Medium-Range Weather Forecasts fifth generation atmospheric reanalysis product) (*80*). White squares indicate sites of prior paleoclimate study or cave monitoring mentioned in the text, with the site of this study, Grutas Tzabnah, marked X. White circles are Northern Maya Lowland sites included in the archaeological data compilation (*5*), with sites mentioned in the text marked with a star (Fig. 4). asl, above sea level.

Our study focuses on resolving the ancient Maya relationship with individual extreme climate events in northern Yucatán (Fig. 1) throughout the Terminal Classic. The historical record (1500 to 1900 CE) (*23*) highlights the region’s susceptibility to severe multiyear droughts, and numerous paleoclimate records suggest that the Terminal Classic was an anomalously dry period within the past two millennia (*12*, *13*, *15*, *16*, *24*–*26*). There exist proxy climate records from the Southern Maya Lowlands that can resolve drought on a seasonal-to-annual basis (*17*, *27*, *28*), but clear seasonality has not yet been observed during the Terminal Classic. In the Northern Maya Lowlands, Medina-Elizalde *et al.* (*15*) reported an approximately annual (1 data point/year between 800 and 940 CE) record from a stalagmite (“Chaac”) collected from the cave Grutas Tzabnah (Fig. 1). Here, we build upon that work by replicating the record using a different stalagmite (“Tzab06-1”) from the same cave, this time at subannual resolution, and with the addition of modern local rainfall and drip water isotope data to support the proxy interpretations. The combined records provide strong evidence for consecutive years of seasonal droughts whose ages and durations are accurately and precisely determined, enabling detailed comparisons with archaeological evidence for cultural change at nearby ancient Maya sites.

## RESULTS

### Stalagmite Tzab06-1

**Fig. 2.**
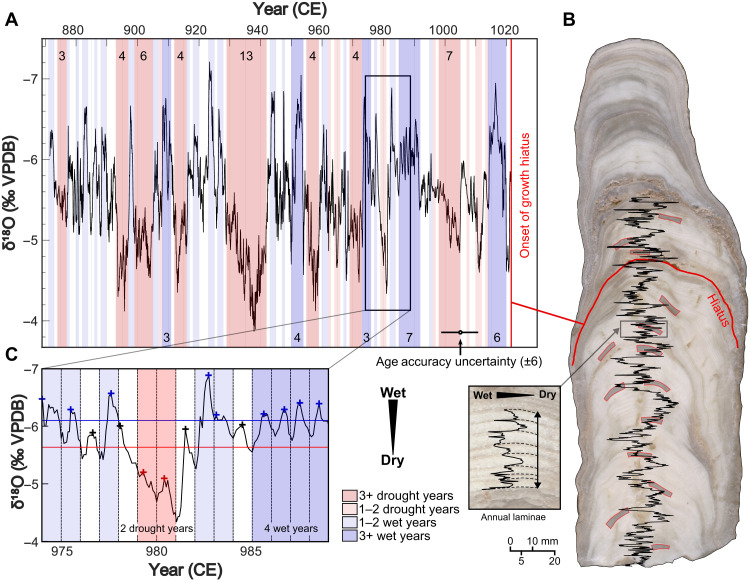
Oxygen isotope results from Tzab06-1. (**A**) The Tzab06-1 δ^18^O record spans the Terminal Classic, with droughts highlighted by red shading, herein defined as periods of 1 to 2 years (pale red) and ≥3 years (dark red), when the minimum (top on inverted axes) annual δ^18^O value was greater than −5.74 per mil (‰). Wet periods are highlighted as blue shading, herein defined as periods of 1 to 2 years (pale blue) and ≥3 years (dark blue), when the minimum (top on inverted axes) annual δ^18^O value was lower than −6.06‰. The record continues above the 45 ± 20–year hiatus that started in 1021 CE, but with poorer age constraint (see Materials and Methods and figs. S1 and S2). (**B**) Tzab06-1, with the hiatus lamina (visible detritus) in red and U-Th date locations in gray (red outlines). Zoomed section: An expanded section of the speleothem showing identified laminae and corresponding δ^18^O, 961 to 969 CE, with more negative values to the left. (**C**) An example of seasonal cyclicity, with events assigned as in (A). Crosses indicate the minimum (top on inverted axes) annual δ^18^O value, colored red (drought), black (average year), or blue (wet year).

Stalagmite Tzab06-1 was obtained in 2006 from Grutas Tzabnah near Tecoh, Yucatán, Mexico (Fig. 1 and text S1). The cave is located near several large Classic Maya sites (most notably Chichén Itzá and many sites in the Puuc Region such as Uxmal) and experienced the same regional climate regime as the major Terminal Classic population centers in northwest Yucatán (*29*). The stalagmite exhibits visible laminations in the section that formed between ~870 and 1100 CE (see Materials and Methods and Fig. 2C). We interpret each lamina as a single year of deposition, which is supported by cyclical variations in δ^18^O and/or δ^13^C, reflecting seasonal differences in rainfall (*30*–*32*) (see Materials and Methods and figs. S1 and S2). We constructed an age model using a floating layer–counting chronology anchored to 15 U-Th disequilibrium ages (see Materials and Methods and Fig. 2).

Annual growth laminae were used to determine precisely the duration of extreme, multiyear rainfall events. We counted 186 annual laminae (with a counting error of +0/−3 years; see Materials and Methods), each ~1 mm in thickness (Fig. 2). The exceptionally high stalagmite growth rate enabled subsampling of each annual layer for stable isotope analysis at a resolution of ~12 sequential powder samples per lamina (2449 total samples; table S1). The record spans the periods 871–1021 and ~1070 to 1100 CE, with a 45 ± 20–year hiatus from 1021 to ~1070 CE. The age model is precise to the year, with an accuracy of ±6 years below the hiatus and ±20 years above (see Materials and Methods). Here, we focus on the record that spans 871 to 1021 (±6 years) CE because of its exceptional chronology and relevance for ancient Maya cultural change.

### Seasonality and meteorological drought identification

The wet season in the Maya Lowlands occurs from approximately mid-May to October (*33*, *34*), when prevailing easterly winds transport moisture sourced from the Caribbean Sea. There is a strong rainfall gradient across the Yucatán Peninsula, from the wetter southeast (1200 to 1600 mm/year) to the drier northwest (300 to 600 mm/year) (Fig. 1) (*29*).

**Fig. 3.**
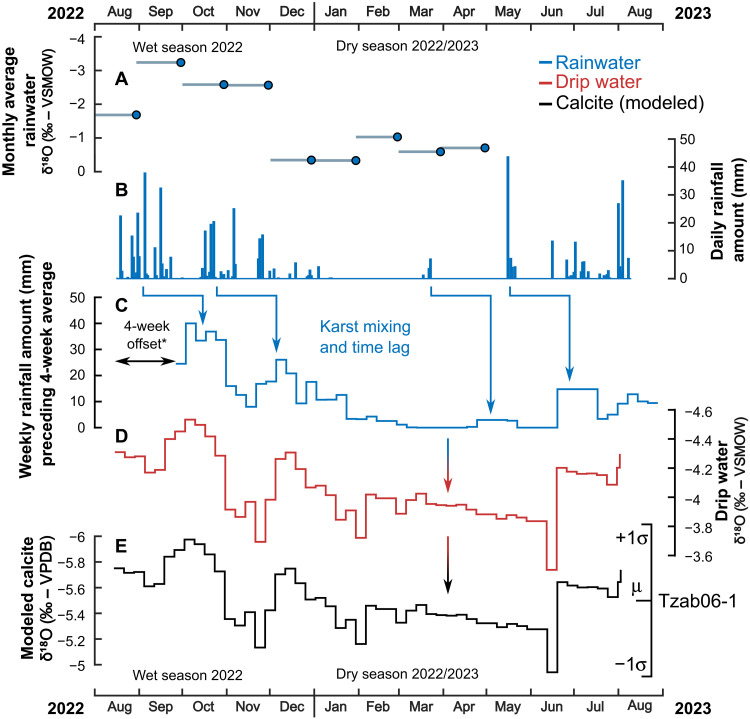
Results of 1 year of rainwater and drip water monitoring at Grutas Tzabnah August 2022 to August 2023. (**A**) Monthly rainwater δ^18^O, with each point averaging the previous calendar month (horizontal lines). (**B**) Daily rainfall amount directly above the cave. (**C**) Weekly rainfall amount, modified to incorporate the effects of mixing and residence time in the karst, modeling the amount of water reaching the roof of the cave. Weekly rainfall was offset by 4 weeks (karst residence time) and averaged over the preceding 4 weeks (mixing during residence). *Averaging over the preceding 4 weeks, not the 2 weeks either side of a given date, is mathematically equivalent to an additional 2-week offset. A residence and mixing time of 4 weeks produces the best match with drip water δ^18^O. (**D**) Drip water δ^18^O, sampled weekly, inherits the variation in rainfall amount (C). (**E**) Modern calcite δ^18^O, forward modeled from δ^18^O_dw_ (D), assuming equilibrium precipitation (see text S4), compared to the mean and ±1σ range of stalagmite δ^18^O_cc_ in Tzab06-1 (right).

We used subannual calcite (δ^18^O_cc_) variations in the Tzab06-1 stalagmite to track wet-season drought, year by year, through the Terminal Classic. Tzab06-1 displays clear sinusoidal δ^18^O_cc_ variability in each of its visible laminae (Fig. 2C and fig. S2). Rainwater and drip water monitoring from 2022–2023 show that Tzabnah rainwater δ^18^O (δ^18^O_rw_) and drip water δ^18^O (δ^18^O_dw_) values are lower during the wet season (approximately mid-May to October, with interannual variability) and higher during the dry season (approximately November to mid-May), with a lag of ~1 month, attributable to the residence time of the infiltrating water in the karst (Fig. 3 and table S2). In addition to seasonal (intra-annual) variability, higher δ^18^O_rw_ values occur during drought years, as shown in multiyear monitoring records of monthly δ^18^O_rw_ at Río Secreto Cave, also located in the Yucatán Northern Lowlands (Fig. 1) (*35*). During the local drought in 2016, mean summer δ^18^O_rw_ at Río Secreto Cave was −2.7 per mil (‰) [Vienna Standard Mean Ocean Water (VSMOW)], substantially higher than values in the previous two nondrought years [−5.0 and −5.3‰ (VSMOW), respectively] (*35*, *36*) (fig. S7). On the basis of the modern rainwater and drip water isotope observations at Tzabnah Cave (our study site) and at Río Secreto Cave, we identified drought events in the Tzab06-1 subannual stalagmite δ^18^O_cc_ record as anomalously high δ^18^O_cc_ values during the wet season, with each lamina’s wet season identified as the lowest δ^18^O_cc_ in the lamina’s annual δ^18^O_cc_ cycle. This interpretation of higher calcite δ^18^O_cc_ being associated with drier conditions is consistent with the previous study of stalagmite δ^18^O_cc_ variations in Grutas Tzabnah (*15*).

The amplitude of the Tzab06-1 seasonal calcite δ^18^O_cc_ cycle within each lamina varies from 0.2 to 1.6‰, which is smaller than the observed 2022–2023 Grutas Tzabnah rainwater δ^18^O_rw_ seasonal amplitude of 3‰ but is comparable to the expected modern δ^18^O_cc_ amplitude, modeled from observed drip water δ^18^O_dw_ (Fig. 3E, 1.0‰). Grutas Tzabnah is a very shallow cave system, which experiences rapid transmission of rainfall (and seasonal δ^18^O variation) to drip water in the cave (quantified to ~1 month; Fig. 3). The overburden above the chamber in which Tzab06-1 grew is only 7 m in thickness, minimizing the time and space available for mixing of rainfall with karst waters (*37*, *38*). Thus, we are confident that Tzab06-1 δ^18^O_cc_ records drought events, despite the muted seasonal signal. The relationship between modern rainfall amount and Grutas Tzabnah δ^18^O_dw_ is clear (Fig. 3) and comparable to that at Río Secreto Cave (*35*, *36*), but the lower seasonal amplitude of δ^18^O_dw_ and δ^18^O_cc_ relative to δ^18^O_rw_ confounds simple application of a modern calibration to estimate absolute changes in paleorainfall [e.g., (*15*, *39*)].

While the exceptionally thin overburden and minimal soil cover at Grutas Tzabnah are ideal for rapid and reliable transmission of δ^18^O_rw_ to the stalagmite, the cave structure strongly inhibits the utility of secondary climate proxies such as trace element content and δ^13^C (fig. S1 and tables S1 and S3). These secondary proxies are controlled by soil and karst processes such as prior calcite precipitation, which only occurs minimally at this site and is likely controlled by factors other than rainfall amount (<30% of Ca precipitated before stalagmite; see text S6 for further results and discussion of additional proxies).

### Agricultural drought severity

Alongside other intensive agricultural practices, the ancient Maya practiced shifting swidden agriculture, in which land was cleared and burned during the dry season and fields were planted in the spring, before the onset of the rainy season. The practice is still used today throughout the region. Crop production and yield are highly dependent on seasonal precipitation, with the amount and timing of rainfall critically important. This is especially true for the main staple, maize (*28*, *40*, *41*). Day-to-day rainfall variability (too much or too little) within a growing season affects crop success or failure, with the timing of cessation of the rains at the end of the wet season being of particular importance (*21*, *41*). In addition, the predictability of annual rainfall patterns (i.e., the expected onset and end of the rainy season) was—and remains today—critically important for Maya farmers, especially in northwest Yucatán where the rainfall gradient is steep, and conditions are generally drier than farther south (Fig. 1). Uncertainty in the timing or amount of rainfall likely increased societal stress for ancient Maya communities already beset by repeated droughts and may have contributed to undermining the authority of the ruling elite (*28*). The ancient Maya developed many drought mitigation techniques such as water storage and irrigation (*42*, *43*) and adoption of more resistant crops such as manioc (*44*–*46*), which were effective at suppressing the effect of droughts up to a threshold severity and duration, where sociopolitical context allowed.

Fedick and Santiago (*44*) defined three levels of drought severity in the Yucatán Peninsula, which affect crops to varying degrees: (i) short-duration drought, i.e., a year with an extended dry season (up to three additional months); (ii) moderate-duration drought, up to a full year of dry season–type rainfall, amounting to a missed wet season; and (iii) extreme drought, i.e., multiple years without normal rainfall or consecutive missed wet seasons. They suggested that during an extreme drought, availability of edible plant species would be reduced by 89% relative to years with normal rainfall. With respect to maize, beans, and squash, which comprise some of the core crops of Maya milpa agriculture, only maize is likely to be productive in short-duration drought, whereas none of these three staple crops is produced in sufficient quantity during moderate-duration or extreme droughts. Adaptation to these effects is hindered by the lack of rainfall predictability associated with droughts (*28*). Distinguishing between these drought categories in a paleoclimate record is important for assessing the impact of climate change on past Maya agricultural yields.

On the basis of this classification (*44*), we defined multiyear extreme drought events in the Tzab06-1 record as times when the δ^18^O_cc_ minimum (summer wet season) was more than one SD greater than the mean annual minimum δ^18^O_cc_ value (above −5.74‰) for three or more consecutive years (laminae). Conversely, we defined exceptionally wet periods as three or more consecutive years in which the δ^18^O_cc_ minimum was at least one SD less than the mean annual minimum δ^18^O_cc_ value (below −6.06‰) (text S7).

## DISCUSSION

We identified droughts in the Tzab06-1 record objectively and placed them on a calendar-year chronology, supported by multiple U-Th ages (table S4). This enabled estimation of the timing and duration of multiple meteorological droughts throughout the Terminal Classic Period. In the 150 years recorded below the growth hiatus, we identified eight wet-season extreme droughts of ≥3-year duration. An extreme drought began in 894 (±6) CE (Fig. 2) and lasted four consecutive years, interrupted by a single wet year, which was followed by another 5 years of wet-season drought. That interval was succeeded by the longest identified extreme drought, which lasted 13 consecutive years, 929 to 942 (±6) CE (Fig. 2) and which was longer than any multiyear drought in local historical records (1500 to 1900 CE, with a maximum drought duration of 10 years) (*23*, *47*, *48*).

**Fig. 4.**
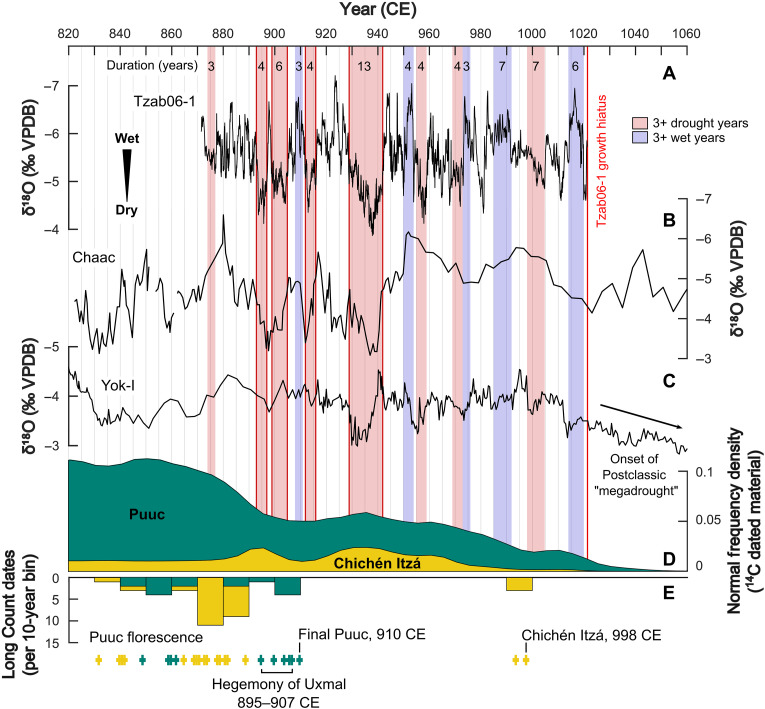
Comparison of Tzab06-1 with regional paleoclimate and archaeological records. (**A**) Tzab06-1 (black) below the growth hiatus, highlighted regions as in Fig. 2, with replicated droughts outlined in red. (**B**) Chaac (*15*), shifted forward 2 years for comparison, within error of their chronology. (**C**) YOK-I (*16*), unshifted. (**D**) Summed probability density functions of Puuc (green) and Chichén Itzá (yellow) radiocarbon-dated archaeological material (*5*). (**E**) Number of Monument Long Count dates (*5*), in 10-year bins and as individual dates [cross symbols, colored as in (D)].

The identified extreme droughts in Tzab06-1 overlap with previously identified dry periods recorded in the lower-resolution Chaac stalagmite δ^18^O_cc_ record from the same cave (*15*) (within uncertainty of their chronologies, shifted 2 years in Fig. 4) and the YOK-I stalagmite record from Belize (*16*) (Fig. 4). Replication of δ^18^O_cc_ patterns in duplicate stalagmites from a single cave, as well as in speleothems from distant caves, supports the interpretation of δ^18^O_cc_ as reflecting changing δ^18^O_rw_ and, ultimately, (pan-)regional changes in effective rainfall, with local variations. Replication among stalagmite records also provides additional support for the accuracy of the chronologies of the individual records (*49*). The seasonal resolution of Tzab06-1 and its precise layer-counting chronology, however, are critical for resolving the timing, duration, relative severity, and seasonality pattern of each drought event. In addition, Tzab06-1 resolves single wet years that fell between longer droughts (e.g., 898 CE), which are likely to be missed in lower-resolution records.

The growth hiatus from 1021 (±6) to ~1070 (±20) CE in Tzab06-1 coincides with the greatest excursion to higher δ^18^O_cc_ values in the YOK-I stalagmite, interpreted as reflecting greatly reduced rainfall in southern Belize (*16*). Depositional hiatuses, such as in Tzab06-1, may occur during droughts when there is insufficient rainfall to sustain the drip, although other factors including changes in drip water chemistry, drip intervals, or migration of the drip location could also suppress calcite precipitation. The period spanned by Tzab06-1 above this hiatus postdates the Terminal Classic and therefore is not discussed further here (text S5). The set of meteorological droughts recorded in the Tzab06-1 and Chaac speleothems is also consistent with the hydrological droughts (*18*) recorded by gypsum horizons in Lake Chichancanab (north-central Yucatán Peninsula), within the large uncertainty of the sediment core chronologies (*12*, *13*) (text S5).

The implications for Maya society of extreme drought events during the Terminal Classic are substantial. Famines caused by drought-induced maize-crop failure in the Colonial Period are estimated to have caused a 20 to 50% loss of the Indigenous Maya population, with the most severe population losses occurring after two droughts between 1648 and 1653 CE (*23*, *50*). Our record reveals the precise duration and relative severity of meteorological droughts that would have affected nearby prehistoric Maya sites as agricultural droughts (*18*). Using both a compilation of radiocarbon dates and Maya Long Count dates inscribed on carved monuments, we compared our drought record to the estimated duration of occupation and societal complexity in the nearby Puuc Region, including the regional capital Uxmal (51 km southwest of Grutas Tzabnah) and Chichén Itzá (94 km east of Grutas Tzabnah) (Fig. 4, D and E).

The Puuc region is both highly fertile and especially vulnerable to water stress (*21*, *32*, *51*). Because of the elevation of the land surface above the water table and lack of accessible permanent water bodies, including cenotes (sinkholes), the Puuc Maya relied on water in aguadas (reservoirs) and chultunes (cisterns) (*42*, *43*). Fluctuations in the availability of this scarce but essential resource, as well as a societal focus on site expansion, led to a boom-and-crash economy that was highly reliant on crop yields (*21*). Radiocarbon dates suggest that Classic Period Puuc Maya construction activity peaked in two distinct pulses, 650 to 800 CE and 850 to 925 CE (*5*). The latest (most recent) calendar dates carved on monuments at sites in the Puuc Hills all fall in the early 900s CE. Uxmal, as a specific example, has hieroglyphic records that span from 895 CE to a terminal Long Count date of 907 CE (Fig. 4E) (*51*). The end of monumental construction and hieroglyphic writing does not necessarily correspond to site abandonment but, instead, likely reflects the decline of Classic Maya political systems (*2*). These terminal dates cluster in three rapidly occurring multiyear droughts recorded in Tzab06-1: 893 to 897 CE, 899 to 905 CE, and 912 to 916 CE, with the final Long Count date in the Puuc (Fig. 4E), occurring 2 years before the third drought, and no multiyear wet period until 922 to 925 CE. We suggest that the recurrence of these drought events and the lack of intervening wet years, which might have been sufficient to ameliorate impacts on the fragile agricultural and political system, were severe enough to cause societal instability and the cessation of monument building (*52*, *53*), although resilient water management practices ensured the polity’s survival (*42*, *52*, *54*). It also seems likely that the predictability of seasonal rainfall suffered from these highly variable hydrological conditions (*28*), which may have undermined confidence in the ruling elites. Even today, declining rainfall predictability remains a key concern for Maya farmers (*55*).

Evidence suggests a short time span (895 to 907 CE) for the apex of hieroglyphic writing at Uxmal, punctuated by multiple extreme droughts, and estimates for the end of the political system there are centered on 950 CE [(*51*, *56*), but see also (*57*)], i.e., 8 years after the most severe recorded drought, which lasted 13 years (929 to 942 CE), with no substantial wet season and no period of consecutive wet years until 986 CE. This likely contributed to a situation in which even elaborate water management became insufficient to support local agriculture and densely populated urban centers (*43*). Evidence from across the wider, ecologically diverse Puuc region echoes this pattern of site abandonment (*5*). Radiocarbon-dated material at the smaller sites of Labna and Kiuic indicates abandonment in the late 9th and early 10th centuries, contemporaneous with or immediately preceding the end of construction at Uxmal (*58*–*60*), although some polities may have remained occupied until as late as the early 1000s CE (*61*), and the occupation chronology of many sites remains a topic of debate.

To the east and at lower elevation than the Puuc Hills, the site of Chichén Itzá responded differently to these environmental stresses. “Old Chichén,” constructed in the Puuc style, according to radiocarbon dates between 605 and 845 CE, declined contemporaneously with these droughts. However, “New Chichén,” built in the “international style,” was able to recover and flourish into the late 900s CE, possibly reliant on trade of maize for more durable resources from central Mexico (*62*, *63*). Terminal Classic Chichén Itzá controlled a vast system of tribute collection that spanned large areas of central and northern Yucatán, a strategy that may have helped mitigate drought impacts (*5*, *50*, *64*). This recovery and florescence occurred during a period of reduced drought frequency and severity between 942 and 1022 CE, which had only three multiyear droughts, with >10 years separating them, and four intervening multiyear wet periods. Perhaps, this was manageable with available water conservation techniques to mitigate crop failures, enabling populations at Chichén Itzá to recover, whereas frequent alternation of wet and dry periods reduced seasonal predictability and prevented reestablishment of less centralized and resilient polities elsewhere (*28*). The decline and collapse of New Chichén are thought to have occurred in the early to mid-1000s CE (1025 to 1050 CE) [(*5*, *65*), but see also (*66*, *67*)], during the “megadrought” recorded in the YOK-I speleothem (*16*) and at Lake Chichancanab (*13*) and the latest estimates of site occupation in the Puuc (*61*). Many aspects of the chronology of Chichén Itzá are areas of active debate [e.g., (*68*, *69*)], and additional evidence may refine or realign these comparisons.

Tzab06-1 provides a precisely dated, seasonally resolved local paleoclimate record that spans the Maya Terminal Classic. From 872 to 1021 CE, eight wet-season (summer) droughts occurred and lasted for three or more consecutive years. They were interspersed with equivalent ≥3-year intervals of consecutive years with normal or enhanced wet-season precipitation. The most severe drought event lasted for 13 consecutive years. The exact duration and frequency of droughts enabled fine-grained comparisons of climate change with local archaeology (e.g., at Uxmal) and prehistoric cultural events in northwest Yucatán. The Puuc boom-and-bust cycles and eventual abandonment, as well as the Chichén Itzá florescence in the late 9th and 10th centuries, coincided approximately with the same series of protracted droughts in the Terminal Classic and Early Postclassic Periods, illustrating differential societal responses (*70*). Nevertheless, even Chichén Itzá was ultimately unable to adapt to an early Postclassic megadrought and experienced decline, although the site was not completely abandoned.

## MATERIALS AND METHODS

### Layer counting and high-resolution sampling

The Tzab06-1 stalagmite was cut in half along the central growth axis. High-resolution micromilling was completed using a New Wave Research Micromill, a sampling system consisting of a video microscope coupled with a drill and a set of computer-controlled motorized stages. During milling, each sequential visible lamina was identified using the video microscope. Visually, the laminae are characterized by intercalated layers of darker (more transparent) dense calcite, and matte white, less dense acicular calcite. Laminae areas without clear visible boundaries were noted for lamina count uncertainty calculations. Overall, laminae thicknesses ranged from 300 to 2800 μm, with an average thickness of 900 μm. The sampling resolution, in the range of 50 to 100 μm, was then chosen for each lamina, such that ~10 to 20 samples were obtained per lamina, depending on thickness and density-controlled carbonate powder yield. Lower-density samples required extraction of greater calcite volume and at least 100 μg of powder was targeted for each sample. Powder samples were collected in a continuous trench along the central growth axis of the stalagmite, or as near to it as possible, avoiding voids. A preliminary trench was milled along one side of the sampling trench to minimize sample overlap error caused by the diameter of the drill bit (*38*). A total of 2449 carbonate samples were collected (Fig. 2, fig. S1, and table S1).

The transition from darker (denser) to matte white (acicular) calcite corresponds with troughs (low values) in the calcite δ^18^O (δ^18^O_cc_) cycles. We interpret δ^18^O_cc_ minima as indicative of wet seasons, which occur during boreal summer on the Yucatán Peninsula (see Results). We suggest that darker denser calcite was precipitated in the wet season (boreal summer) under a rapid drip, whereas the void-rich, needle-like calcite was precipitated in the dry season (boreal winter) under lower drip rates and enhanced CO_2_ degassing from the drip water, promoting carbonate deposition (*71*, *72*). The calendar year transition (December to January) was therefore placed halfway between dark layers (δ^18^O_cc_ troughs), amidst the acicular less-dense layers (δ^18^O_cc_ peak, dry season).

After δ^18^O and δ^13^C analysis, the correspondence between sinusoidal isotope patterns and clear visible lamina boundaries was checked, and external reproduction of the layer count was conducted. If a sinusoidal pattern was not identified in δ^18^O and/or δ^13^C of a lamina in one or more layer counts, then a “1-year missing” counting error was attributed to that lamina. Laminae #126, #142, and #149 (all within the same 12-mm section of the stalagmite) each have a 1-year missing counting error (fig. S2). Thus, the total counting error is +0/−3 years, and the total number of years spanned by all laminae, with counting error, is 183 to 186 years, with a modal value across recounts of 186 years, the value used for tethering to U-Th tie points.

### Stable isotopes

All 2449 calcite samples were analyzed for δ^18^O_cc_ and δ^13^C_cc_ values at the Godwin Laboratory, University of Cambridge, using a Thermo Fisher Scientific Delta V isotope ratio mass spectrometer coupled to a GasBench II on-line gas preparation/introduction system. For each sample, ~100 to 150 μg of carbonate was sealed in a borosilicate glass Exetainer vial with a silicone rubber septum and loaded into the Thermo Fisher Scientific GasBench autosampler that holds 40 samples. Each batch of samples included 10 reference carbonates of the in-house standard Carrara Z [calibrated to Vienna Pee Dee belemnite (VPDB) using the international standard NBS19] and two control samples of Fletton clay. Samples and standards were first flushed with helium, then acidified with 104% orthophosphoric acid for 1 hour at 70°C, and analyzed with the Thermo Fisher Scientific Delta V mass spectrometer in continuous flow mode. Precision of Carrara Z (δ^18^O = −1.27‰ and δ^13^C = 2.25‰) was ±0.06 ‰ (1σ) or better for δ^18^O_cc_ and δ^13^C_cc_.

### U-Th disequilibrium dates

Calcite for U-Th age samples was milled using a New Wave Research MicroMill (the same system used for high-resolution stable isotope sampling). U-Th sample trenches spanned no more than three visual laminae and yielded 150 to 200 mg of calcite powder, sufficient to minimize instrumental uncertainty relative to counting statistics. Sample preparation and chemical analyses were conducted at the University of Oxford. Samples were weighed (three times), dissolved in concentrated nitric acid (HNO_3_, 16 M), and spiked with a mixed ^229^Th/^236^U solution to enable correction for imperfect yield during column chemistry. Spike mass was chosen to best match spiked sample ^229^Th/^230^Th to known in-house ^229^Th/^230^Th solution standards.

U and Th were separated by column chemistry using procedures adapted from prior methodology (*73*). Acid-leached poly-prep chromatography columns [polypropylene, 9 cm high, 2-ml bed volume (0.8 cm by 4 cm), and 10-ml reservoir] were used with acid-cleaned AG 1-X8 Anion Exchange Resin, analytical grade, 100 to 200 mesh, in chloride form.

U and Th isotopes were measured with a Nu Plasma II multi-ion counting, multicollector inductively coupled plasma mass spectrometer, following adapted procedure (*74*). U measurements were performed using Faraday cups for ^238^U, ^236^U, and ^235^U and an ion counter for ^234^U. Th measurements were performed using Faraday cups for ^232^Th and ion counters for ^229^Th and ^230^Th. Abundance sensitivity in U (Th) measurement was corrected on the basis of the half-mass measurements at 234.5 and 233.5 (230.5, 229.5, and 228.5) on the ion counter after each sample measurement. Machine biases were corrected using sample-standard bracketing with CRM-145 for U and two in-house ^229^Th-^230^Th-^232^Th standards for Th.

Ages were calculated iteratively using the ^230^Th/^238^U age equation of Kaufman and Broecker (*75*), derived by Richards and Dorale (*76*), using the half-lives reported by Cheng *et al.* (*77*). Age uncertainty was calculated using a Monte Carlo method, with each iteration choosing from a normal distribution of ^234^U/^238^U, ^230^Th/^238^U, and ^232^Th/^238^U ratios to solve the age equation. These ratios were calculated from instrument-measured ^234^U/^236^U, ^238^U/^236^U, ^230^Th/^229^Th, and ^232^Th/^229^Th using the isotope dilution analysis equations for known ^229^Th/^236^U spike concentrations. ^234^U/^238^U, ^230^Th/^238^U, and ^232^Th/^238^U errors were therefore calculated using a Monte Carlo method that propagated sample isotope ratio instrument error, spike isotope ratio error, and chemistry blank error. Initial errors on the instrument-measured ratios were calculated by propagating SE (precision) calculated over multiple measurement cycles and comparison with secondary standards (accuracy).

### U-Th age correction for initial Th

The measured ^230^Th/^232^Th activity of the 15 U-Th samples ranged from 48 to 96 (table S4), suggesting substantial initial Th contamination in the samples, which required a correction for accurate age calculations (*78*). We used the number of years between each milled date, based on the lamina counts and their associated uncertainty, as a priori knowledge to determine a best-estimate initial ^230^Th/^232^Th range to calculate corrected U-Th ages (table S4). A Monte Carlo simulation (*n* = 10,000) of varying initial ^230^Th/^232^Th atomic ratios was propagated through the age equation in bins with a width of 5 × 10^−6^. Results (fig. S3) show that the difference between corrected U-Th ages and lamina counts is minimized in the atomic ratio range of 25 × 10^−6^ to 35 × 10^−6^. This corresponds to an activity ratio range of 4.6 to 6.5. A uniform distribution in this range was propagated throughout the subsequent age model, and the associated uncertainty was incorporated in all resultant age errors.

### Age model

Annual δ^18^O (and δ^13^C) cyclicity was tuned to calendar years by aligning δ^18^O (and δ^13^C) maxima with the December to January calendar boundary [as in, e.g., (*79*)]. This relies on the assumption that δ^18^O_cc_ (and δ^13^C_cc_, which varies in phase; text S6) cycles annually, with maxima in the dry season. The precise timing of the dry season extreme will vary year to year, and, hence, the identified years are mid-dry season to mid-dry season, not strictly January to December. This adjustment did not alter the number of years recorded.

Floating layer count age models were anchored to calendar years using the corrected U-Th disequilibrium dates (table S4 and figs. S2 and S3). A horizon of granular detrital material is apparent between 150 and 151 laminae from base (Fig. 2) and is identified as a depositional hiatus. Hence, the Tzab06-1 age model was constructed using two floating layer counts of continuous growth: laminae #1 to 150 (150 years, tethered using 12 U-Th ages, of which two are replicates on the same growth layers) and laminae #151 to 186 (35 years, tethered using 3 U-Th ages).

Each floating layer count age model was anchored to calendar ages by fitting the floating age model with its associated uncertainty through the highest possible number of U-Th age 68% confidence intervals and all U-Th age 95% confidence intervals. In the older section, the age models that fulfilled these requirements have a starting date range of 866 to 878 (872 ± 6) CE (fig. S4). In the younger section, the age models that fulfilled these requirements have a starting date range of 1047 to 1087 (1067 ± 20) CE. Hence, we estimated the age uncertainty in the older section as ±6 years and the age uncertainty in the younger section as ±20 years. The date ranges of the two tethered age models further constrain the start and end date ranges of the growth hiatus: beginning 1021 ± 6 CE and ending 1067 ± 20 CE.

The U-Th date ascribed #144 laminae from base lies above two of the laminae with 1-year missing lamina count errors, and therefore its position lies in the range of #142 to 144 laminae from base. Within this range, #144 (±0) is the position resulting in the largest uncertainty for the final tether (fig. S5) and, hence, is taken as the position for the purpose of maximizing uncertainty. No other dates are affected by the lamina count error, as the tether above the hiatus is independent of count below (and its associated error), and, hence, the tethering methodology is not influenced by counting error in this case.

In the region of the stalagmite containing the three laminae with associated counting uncertainty (135 to 147 mm from base, 997 to 1021 CE, ending at the hiatus), the +0/−3-year counting error is combined with the ±6 tethering error for a total error of +6/−7 years by established methods (*32*), and, hence, the hiatus strictly begins in 1021 +6/−7 CE.

### Cave monitoring

Rainfall amount was recorded between August 2022 and August 2023 at Grutas Tzabnah using a HOBO Rain Gauge Data Logger (RG3, Onset Computer Corp.) coupled to a 5-liter rainwater collection container. The container was sampled and emptied on the first day of every calendar month. Drip water was collected using a SYP (Waikato Scientific Instruments, waikatoscientific.com) autosampler, beneath an active drip in the region most likely to have hosted Tzab06-1 (fig. S6). The SYP collected the first 15 ml of drip water to accumulate in each 7-day interval (August 2022 to August 2023) in 15-ml sealed polypropylene sampling vials. Internal cave temperature was measured using a TinyTag Plus-2–TGP-4500 logger (Gemini Data Loggers).

Water isotopes were analyzed at the Godwin Laboratory for Palaeoclimate Research (University of Cambridge) using an L2140-*i* Picarro water isotope analyzer coupled to an A0211 high-precision vaporizer (Picarro, Santa Clara, CA, USA). Samples were normalized against VSMOW and drift corrected versus three internal standards: BOTTY (δ^18^O = −7.4‰, δD = −50.4‰, *n* = 35), SPIT2 (δ^18^O = −0.15‰, δD = −0.44‰, *n* = 9), and DY2 (δ^18^O = −23.1‰, δD = −180.5‰, *n* = 9).

## Supplementary Materials

### The PDF file includes:

Supplementary Text Figs. S1 to S14 Legends for tables S1 to S4 Table S5 Example Analysis Script References
