# Supplementary material for: Classic Maya response to multiyear seasonal droughts in Northwest Yucatán, Mexico
Source: Sci Adv. 2025 Aug 13;11(33):eadw7661. doi: 10.1126/sciadv.adw7661 (PMC12346346; doi:10.1126/sciadv.adw7661)
Supplement: Supplementary file 1 — Supplementary Text Figs. S1 to S14 Legends for tables S1 to S4 Table S5 Example Analysis Script References [file sciadv.adw7661_sm.pdf]

Science Advances
AAAS

Supplementary Materials for

# Classic Maya response to multiyear seasonal droughts in Northwest Yucatán, Mexico

Daniel H. James *et al.*

Corresponding author: Daniel H. James, daniel.h.james@ucl.ac.uk

*Sci. Adv.* **11**, eadw7661 (2025)
DOI: 10.1126/sciadv.adw7661

**The PDF file includes:**

Supplementary Text
Figs. S1 to S14
Legends for tables S1 to S4
Table S5
Example Analysis Script
References

**Other Supplementary Material for this manuscript includes the following:**

Tables S1 to S4

# Supplementary Text

## S.1 Specimen Information

### S.1.1 Field Sampling

Grutas Tzabnah, which means "King's Palace" in Maya, is located 40 km SE of Mérida near the town of Tecoh, Yucatán, Mexico. The Tzab06-1 speleothem was collected in 2006. With consideration of cave conservation, the analysed stalagmite was selected from previously broken and harvested material. The most probable stalagmite base was identified during fieldwork in 2022. This sample location is in a large chamber ("Dome of the Cathedral") located ~425 m from the entrance, beneath ~7 m of overburden, and contains both standing water (Cenote 10) and a small artificial well (La Noria) in the chamber ceiling, constructed in the Colonial Period (fig. S6).

Initial lower-resolution $\delta^{18}O$ and U/Th analysis was conducted on Tzab06-1 in 2007 to identify its palaeoclimate utility. Those preliminary data are not presented, and all U/Th and stable isotope data herein were collected in 2021 and 2022. The sampled section of Tzab06-1 was stored at room temperature at the University of Florida and University of Cambridge between 2006 and recent analysis starting in 2021.

### S.1.2 Provenance

Tzab06-1 was retrieved from Grutas Tzabnah by the cave's custodians before 2006 and gifted to David A. Hodell in 2006 in collaboration with representatives from Tecoh's local government, which owns and manages the cave, and with permission from the *Instituto Nacional de Antropología e Historia (INAH)*. As part of this study, Tzab06-1 was validated for authenticity by replication with another stalagmite from the same cave (*Chaac*) and dated extensively (Materials and Methods). Tzab06-1 is held by David A. Hodell at the University of Cambridge and is accessible upon request.

## S.2 Local Climate and Stable Isotopes of Rain

At Grutas Tzabnah (Fig. 3) and Río Secreto (fig. S7), the oxygen isotope ratio of rainwater ($\delta^{18}O_{rw}$) captures the annual cycle of rainfall amount, with more negative values in the wet season (boreal summer) and less negative values in the dry season (boreal winter). This demonstrates the ability of $\delta^{18}O_{rw}$ in the northern Yucatán region to record seasonal cyclicity, though (palaeo)climate evidently varies on a local scale, such as between Grutas Tzabnah and Río Secreto.

The multi-year climate dataset at Río Secreto recorded the local meteorological wet-season drought year of 2016 (May-October 2016 rainfall was 466 mm, versus that at the nearby Playa del Carmen meteorological station, with a long-term average of 950 mm), with amount-weighted summer $\delta^{18}O_{rw}$ -2.7 ‰ (VSMOW), compared with the previous two years, which had amount-weighted summer $\delta^{18}O_{rw}$ values of -5.0 ‰ and -5.3 ‰ VSMOW (*35,36*)

(fig. S7). We expect that Grutas Tzabnah will be able to record seasonal drought in the same way, when multi-year monitoring data become available.

## S.3 Stable Isotopes of Dripwater

The single year of monitoring data at Grutas Tzabnah indicate that the seasonal signal in $\delta^{18}O_{rw}$ is reliably transferred to the oxygen isotope ratio of dripwater ($\delta^{18}O_{dw}$). Fig. 3 (main text) shows the single full year of data, which displays a distinct seasonal cycle with lower $\delta^{18}O_{dw}$ during the wet season and higher $\delta^{18}O_{dw}$ during the dry season, like the $\delta^{18}O_{rw}$ signal. This seasonality is also apparent in δD and D-excess (fig. S8). These data confirm our assertion that the thin (~7 m) karst overburden atop Grutas Tzabnah does not cause a reservoir-effect sufficient to eliminate the seasonal $\delta^{18}O_{rw}$ signal, although the amplitude of intra-annual variability is damped.

Direct comparison of rainfall amount and $\delta^{18}O_{dw}$ reveals inheritance of monthly-scale variation in rainfall amount by $\delta^{18}O_{dw}$, with an offset of one month, to the nearest month, (Fig. 3, main text). Comparison of $\delta^{18}O_{dw}$ and $\delta^{18}O_{rw}$ corroborates this: regression between the two measures with no offset yields $R^2 = 0.23$, a one month offset increases the regression to $R^2 = 0.51$, a two-month offset then decreases it to $R^2 = 0.03$, and >2 months produces a negative correlation.

## S.4 Stable Isotopes of Modelled Modern Calcite

We expected the variation in $\delta^{18}O_{dw}$ to be inherited by the oxygen isotope ratio of precipitated calcium carbonate ($\delta^{18}O_{cc}$). We used a simple forward model to construct expected equilibrium $\delta^{18}O_{cc}$ based on sampled $\delta^{18}O_{dw}$ at Grutas Tzabnah for comparison to Tzab06-1 (Fig. 3, main text).

To forward model $\delta^{18}O_{cc}$ from $\delta^{18}O_{dw}$, we used a best-estimate approximation of the equilibrium fractionation factor of oxygen isotopes between calcite and water at low temperatures. Equation S1 relates the calcium carbonate-dripwater (cc-dw) fractionation factor ($\alpha_{cc\text{-}dw}$) of $^{18}O$ to temperature (T, Kelvin), based on a collection of modern cave measurements that span a range of temperatures (*81*). We set T = 300.35 K, the stable annual temperature of the chamber in which Tzab06-1 grew (27.2 °C, August 2022 to August 2023).

$$10^3 ln\alpha_{cc-dw} = 16.1 * \left(10^3/_T\right) - 24.6 \qquad \text{(S1)}$$

Equations S2 and S3 are the definitions of $\alpha_{cc\text{-}dw}$ and $\delta^{18}O_{dw}$ respectively, here using the VSMOW seawater reference, where R is the isotope ratio $^{18}O/^{16}O$ in the relevant phase (*82*). Given the known standard $R_{VSMOW} = 2.0052\times10^{-3}$, and $\alpha_{cc\text{-}dw}$ calculated above, Equations S2 and S3 can be rearranged to model $R_{cc}$ from measured $\delta^{18}O_{dw}$ (Equation S4) (*83*):

$$\alpha_{cc-dw} = \frac{R_{cc}}{R_{dw}} \quad \text{(S2)}$$

$$\delta^{18}O_{dw,VSMOW} = \left(\frac{R_{dw}}{R_{VSMOW}} - 1\right) * 1000 \quad \text{(S3)}$$

$$R_{cc} = \alpha_{cc-dw} * \left({\delta^{18}O_{dw,VSMOW}}/{1000} + 1\right) * R_{VSMOW} \quad \text{(S4)}$$

Rcc can then be converted to $\delta^{18}O_{cc}$ in permille notation by Equation S5, relative to the VPDB carbonate reference, for which the known standard $R_{VPDB} = 2.067 \times 10^{-3}$:

$$\delta^{18}O_{cc,VPDB} = \left(\frac{R_{cc}}{R_{VPDB}} - 1\right) * 1000 \quad \text{(S5)}$$

The forward-modelled $\delta^{18}O_{cc}$ is shown in Fig. 3 (main text). The seasonal amplitude in forward-modelled $\delta^{18}O_{cc}$ (-5 ‰ to -6 ‰ VPDB) falls within both the range of values recorded in Tzab06-1 (-7 ‰ to -4 ‰) and the window ±1σ from the mean (-5.5±0.60 ‰), indicating both equilibrium precipitation, and stable modern-like cave conditions during the period when Tzab06-1 was accumulating. Calcite farming at Grutas Tzabnah is required to confirm these relationships, including the specifics of the water-calcite relationship there and the possible influence of non-equilibrium effects (*84,85*).

## S.5 Comparison to other records

### S.5.1 Palaeoclimate Proxy Records

In comparing Tzab06-1 to other palaeoclimate records from the region (Fig. 4, main text), we prioritised the chronology of Tzab06-1, given its smaller uncertainty. For comparison, *Chaac* was shifted forward by two years, well within the stated uncertainty of the record (*15*) (±10). No alterations were made to the chronology of the southern Belize Yok-I speleothem (*16*).

The additional local speleothem record VP-10-01 (*26,86*) displays the same variability as the *Chaac* record, but with lower resolution and chronological certainty. It therefore also corroborates the results from Tzab06-1 (fig. S9).

Sufficiently long meteorological droughts can lower water tables and affect the hydrologic budgets of lakes. Gypsum horizons in sediment profiles from Lake Chichancanab, north-central Yucatán Peninsula, are indicative of *hydrologic* drought, i.e., times when evaporation exceeds precipitation for multiple consecutive years, given that the residence time of lake water is significantly longer than an annual cycle (*87*). This results in lake-level lowering and supersaturation of the lake water with respect to gypsum (*13,87*). We hypothesise that the ≥3-year *extreme droughts* recorded as $\delta^{18}O_{cc}$ excursions in the Tzab06-1 speleothem reflect meteorological drought events of sufficient intensity to induce hydrological drought.

The chronology of the Chichancanab gypsum record is poorly resolved compared to that of Tzab06-1, given the relatively large errors of the $^{14}C$ dates on samples from the lake sediment core. Dates that constrain gypsum deposition have errors on calendar dates that average ±120 years (95 % confidence interval). Additionally, the age model assumes linear interpolation

between each $^{14}C$ tie-point, without accounting for likely different accumulation rates of the interbedded gypsum and organic-rich sediment (gyttja). Although the radiocarbon chronologies of Lake Chichancanab sediment cores are not precise enough to prove temporal correlation between the multi-year droughts recorded in Tzab06-1 and gypsum layers found in the lake, patterns between the two records are similar, under the assumption that only *extreme droughts* are of sufficient intensity and duration to induce hydrological drought (fig. S9). The four multi-year droughts observed between 889 and 938 CE correspond to a set of gypsum peaks in the lake sediment record. Longer droughts (889-894 CE, 895-901 CE, and 925-938 CE) induced thicker gypsum layers, interposed by a thinner layer that might reflect the shorter drought of 908-912 CE, suggesting that the single wet year between the droughts of 889-894 CE and 895-901 CE would not have been sufficient to end the hydrological drought. The later set of gypsum horizons that overlie this initial set may therefore correspond to the 'Postclassic Megadrought' in the YOK-I speleothem (*16*) and the hiatus in Tzab06-1, though the cause of drip cessation at that time is unclear and may not have been associated with dry conditions in Grutas Tzabnah.

S.5.2 Archaeological Records

The radiocarbon probability density function curve plotted in Figs 2 and 4 (main text) is taken from the 2016 study of Hoggarth *et al.* (*5*, Appendix A therein). Spatial coverage of radiocarbon data (which passes quality control criteria) across the Puuc is limited to a small number of sites, particularly Xkipché (27 dates), Chac Il (22 dates), and Xcoch (17 dates), with fewer from Uxmal (9 dates) and only 5 dates from other sites. Until further study is conducted this coverage necessarily biases the compilation and care should be taken drawing conclusions from the chronology beyond the general trend of decline across the period covered by Tzab06-1.

The same sampling bias is unlikely to affect Long Count dates, though it should not be assumed that presence of these inscriptions necessarily implies cultural hegemony, nor that cessation of inscription implies site abandonment. Additionally, Long Count dates are rare in the Puuc region compared to other parts of the Maya Lowlands. Dated monuments do however provide the best opportunity for comparison with Tzab06-1 given then lack of quantifiable age uncertainty. The dates plotted in Fig 4 are as in table S5, being all the Long Count dates quoted in Appendix D in the study by Hoggarth *et al.* (*5*) between 820 and 1000 CE from the Puuc region and Chichén Itzá.

## S.6 Additional Proxies

Carbon isotopes ($\delta^{13}C$) in speleothems can be influenced by changes in climate but also respond to a subset of associated factors. The primary source of carbon to a karst drip is dissolved inorganic carbon (DIC) from respiration in the overlying soil, and as such, $\delta^{13}C$ values reflect the source of organic matter in soil (C3 vs C4 vegetation) and microbial activity in the soil (*88*). In a stable location that is not affected by substantial changes in vegetation type, more productive soils should result in lower DIC $\delta^{13}C$ values in soil water, a correlation that has been used regionally to reconstruct climate (*27,89,90*). In this sense, DIC $\delta^{13}C$ values in soil water covary with $\delta^{18}O_{dw}$ to a degree because the soil is less productive during more arid periods and responds to the seasonal rainfall cycle. We thus would expect to see pronounced seasonality in the speleothem $\delta^{13}C_{cc}$ record, and long-term (decadal-scale) shifts in response to prolonged droughts. Longer droughts would be expected to cause greater soil degradation, as soils cannot be replenished over many consecutive muted wet seasons, as opposed to a single dry year or droughts of only 2-3-year duration. $\delta^{13}C_{cc}$ also responds to calcite precipitation in air-filled voids along the drip pathway in the karst (prior calcite precipitation, PCP). With such a thin overburden, the effect of PCP at Grutas Tzabnah is thought to be minimal (see below).

Seasonal cyclicity in Tzab06-1 is more pronounced for $\delta^{13}C_{cc}$ than for $\delta^{18}O_{cc}$ (fig. S1), with clear maxima and minima visible even during periods of suppressed annual extremes in the $\delta^{18}O_{cc}$ signal. On a decadal scale, three positive $\delta^{13}C_{cc}$ excursions are apparent: c. 880-900 CE, c. 925-945 CE, and c. 970-990 CE. Between c. 970 and 990 CE, the frequency of shorter-duration (1-2 year) dry events appears to have had a substantial impact on soil productivity above Grutas Tzabnah.

In previous studies, investigators chose to analyse $\delta^{13}C_{cc}$ as a regional palaeoclimate proxy, instead of $\delta^{18}O_{cc}$ (*17,27*), given the disproportionate impact on $\delta^{18}O_{cc}$ values of tropical cyclones (with highly negative $\delta^{18}O_{rw}$ and $\delta^{18}O_{cc}$ values) and changes in moisture source (*91*). Our method of age modelling minimises the impact of these factors insofar as the prior layer count ensures that significant negative excursions that occurred over a single year (e.g., from frequent summer cyclones) are not misinterpreted as multi-year wet periods. For example, the $\delta^{18}O_{cc}$ excursion in 898 CE (fig. S1) likely represents a season of more frequent or proximal cyclones amidst a drought, (and is within chronological error of the flooding episode in 896 CE recorded by Frappier *et al.* (*92*)), not a prolonged return to wetter conditions.

Additionally, given the short duration of our record and lack of step changes in $\delta^{18}O_{cc}$, we suggest that there were no significant changes in summer/winter moisture source throughout the covered period. The (volumetric) majority of rain in northern Yucatán is sourced from the Caribbean (*33*), with occasional Gulf of Mexico-sourced storms (*nortes*) during the dry season (*93*). We believe that the monitored correspondence between rainfall amount and $\delta^{18}O_{dw}$ (Fig. 3) demonstrates sufficiently that rainfall amount, not these seasonal changes in moisture source, is the primary component of seasonal $\delta^{18}O_{dw}$ cyclicity here, and $\delta^{18}O_{cc}$ remains a valid proxy for rainfall amount.

Mg/Ca in stalagmites is widely applied as an indicator of in-karst degassing and prior calcite precipitation (PCP). Given that the degree of PCP will be higher when the karst is less saturated, it can be used as a climate (precipitation) proxy on the timescale of karstic infiltration. Stalagmite Mg/Ca is a robust indicator of PCP if the initial Mg/Ca in the dripwater is constant overtime, there is little variation in the partition coefficient of Mg in calcite, and the analysed Mg is precipitated in calcite and not attached to detrital materials. Sr/Ca, while useful, is less robust as a PCP indicator, as the partition coefficient of Sr in calcite may increase with increased growth rate or increasing Mg/Ca ratio (*94-96*).

To investigate these proxies in Tzab06-1, LA-ICP-MS analysis was conducted at Durham University (Thermo Scientific X-Series2 (ICP-MS) coupled with a Teledyne Analyte Excite+). Trace element ratios (cps/cps) for Mg/Ca and Sr/Ca were obtained as continuous profiles (1-3 mm offset from the milled central axis) using a 285x12-μm rectangular slot at 10 μm $s^{-1}$ and a 15-Hz laser repetition rate, resulting in 10-μm spatial resolution.

After removal of outliers, Tzab06-1 Mg/Ca varies around a mean of 8.2±1.8 ($x10^{-3}$, 1σ), and Sr/Ca varies around a mean of 0.41±0.05 ($x10^{-3}$, 1σ), with little interannual variability (fig. S10). Notably, strontium content, and the amplitude of variation, are sufficiently low to rule out seasonal changes in mineralogy across laminae (calcite/aragonite) (*95-98*).

Comparison of $\delta^{13}C$ and Mg/Ca in Tzab06-1 reveals the extent to which PCP controls $\delta^{13}C$. fig. S11 shows Tzab06-1 Mg/Ca and Sr/Ca plotted alongside $\delta^{13}C$, with a consistent 5 mm moving average applied to all records to eliminate sub-annual variability. As seen in fig. S11, for a proportion of the record, interannual variations in $\delta^{13}C$ are correlated with Mg/Ca, and PCP is therefore the dominant control on $\delta^{13}C$ at those times. The Sr/Ca record does not correlate well with $\delta^{13}C$.

Based on these results, we next calculated quantitative PCP variability for the time intervals in which the coupled Mg/Ca and $\delta^{13}C$ records suggest dominant PCP control. PCP is quantified as the proportion of Ca remaining in drip water solution relative to the initial concentration ($f_{Ca}$). Assuming a constant, very low calcite Mg partition coefficient (~$1x10^{-2}$ (*96*)), the simple Rayleigh equation that relates the fraction of initial Ca remaining in solution to the initial drip water Mg/Ca and measured Mg/Ca solution at the time of stalagmite formation is reduced to Equation S6 (*99*):

$$f_{Ca} = {Mg/Ca_{initial}} \Big/ {Mg/Ca_{stalagmite}} \qquad \text{(S6)}$$

Setting the $MgCa_{initial}$ equal to the lowest $MgCa_{stalagmite}$ value allows $f_{Ca}$ to be calculated, such that the highest $f_{Ca} = 1$. The approximate $f_{Ca}$ calculated from Tzab06-1 Mg/Ca, plotted against the robustly tested climate proxy from Tzab06-1, $\delta^{18}O$, is shown in fig. S12.

The extent of PCP was calculated to be small, and to vary less than ±10 % throughout the period studied. Furthermore, in this low-variability range, there does not appear to be a strong correlation between the amount of PCP and rainfall amount, as inferred from the $\delta^{18}O$ record. We are most confident in the stalagmite $\delta^{18}O$ record interpretation, as interannual $\delta^{18}O$ variations are replicated in a second stalagmite from the site (*15*), interannual monitoring data from 2014-2018 confirm wet-season droughts in the Yucatán are reflected in rainfall $\delta^{18}O$

(*35,36*) (fig. S7), and our initial monitoring at Grutas Tzabnah (2022-2023) demonstrates a correlation between rainfall amount and drip water $\delta^{18}O$, with an average offset of one month (Fig. 3). That we record apparent $\delta^{18}O$ variability, but limited PCP variability, is consistent with the environmental variables at this cave site. Grutas Tzabnah is overlain by thin overburden (7 m) and little soil, with common exposure of surface bedrock, both of which enable rapid transmission of rainwater and associated geochemical signals (i.e. $\delta^{18}O$) to the cave but reduce the relative sensitivity of karst processes (i.e. PCP) to changes in rainfall. For this reason, primary proxies ($\delta^{18}O$) are preferred to secondary proxies ($\delta^{13}C$, trace elements) at this location as paleohydrology indicators.

## S.7 Assignment of Droughts

Throughout the $\delta^{18}O_{cc}$ record, droughts were assigned when the minimum value (considered the wettest extreme) within a given year did not drop below a given threshold for three or more consecutive years (fig. S13). Conversely, a 'wet period' was assigned when the minimum $\delta^{18}O_{cc}$ value within a given year did not rise above a given threshold for three or more consecutive years. Both assignments were based on wet season extremes only, as this is the metric that agricultural productivity most strongly responds to, with little regard for the relative severity of dry seasons.

Fedick and Santiago (*44*) defined *extreme droughts* as 'multiple years' of skipped wet seasons, we here increase this to three or more years. The thresholds are defined at 1σ above and below the mean $\delta^{18}O_{cc}$ record of the distribution of wet season extremes (fig. S14), such that extreme droughts or wet events represent consecutive years outside the distribution's 68 % interval. The resultant thresholds are -5.74 ‰ for droughts and -6.06 ‰ for wet periods.

Given that more of the time span of Tzab06-1 is characterised by droughts than wet periods, the $\delta^{18}O$ mean is higher than if the record were longer and included more 'background climate.' Because of this, some marginal years not assigned as droughts here may reflect drier-than-average conditions over longer timescales, and Tzab06-1 may underestimate the long-term relative severity of the assigned droughts.

# Supplementary Figures

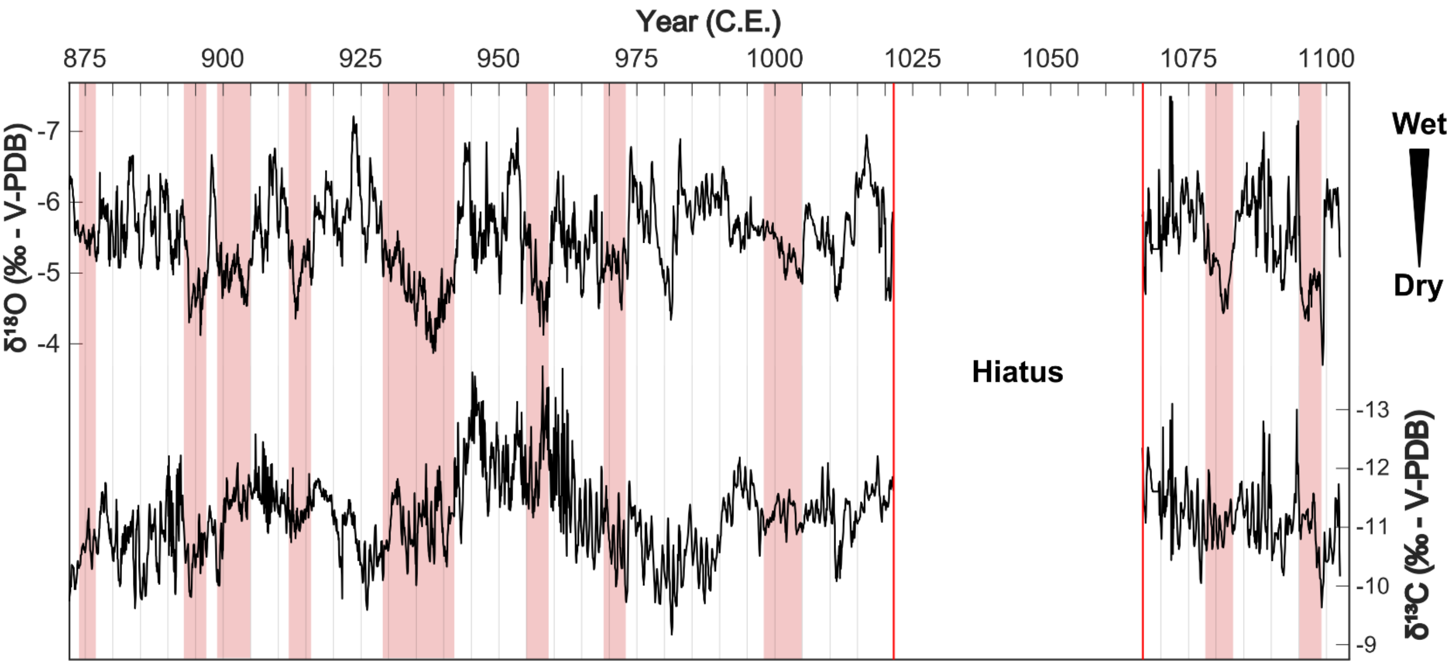


***Fig. S1. Full Record of Tzab06-1 $\delta^{18}O_{cc}$ and $\delta^{13}C_{cc}$.*** *The $\delta^{18}O_{cc}$ and $\delta^{13}C_{cc}$ records from Tzab06-1, in full. Red bars are drought periods (≥3yrs duration), and the area bounded by red is the hiatus. N.B. the age error is ±6 years before the hiatus and ±20 years after the hiatus.*

***Next Page: Fig. S2. Tzab06-1 $\delta^{18}O_{cc}$ and $\delta^{13}C_{cc}$ at fine scale.*** *The $\delta^{18}O_{cc}$ and $\delta^{13}C_{cc}$ records from Tzab06-1 before the hiatus, expanded to highlight annual cyclicity. All annual layers are clear in at least one of the following metrics: visual lamina expression, $\delta^{18}O_{cc}$, or $\delta^{13}C_{cc}$. The red dotted line at -5.74 ‰ $\delta^{18}O_{cc}$ is the threshold for wet-season drought assignment (>1σ less negative than the wet-season mean), and red vertical bars are periods of ≥3 consecutive years in which $\delta^{18}O_{cc}$ remained less negative than the threshold year-round. Grey vertical bars are the three laminae which were not unanimously counted during external layer count reproduction, in each case one recount combined these laminae with an adjacent one (998 and 999 CE, 1012 and 1013 CE, and 1020 and 1021 CE), generating the +0/-3 count error.*

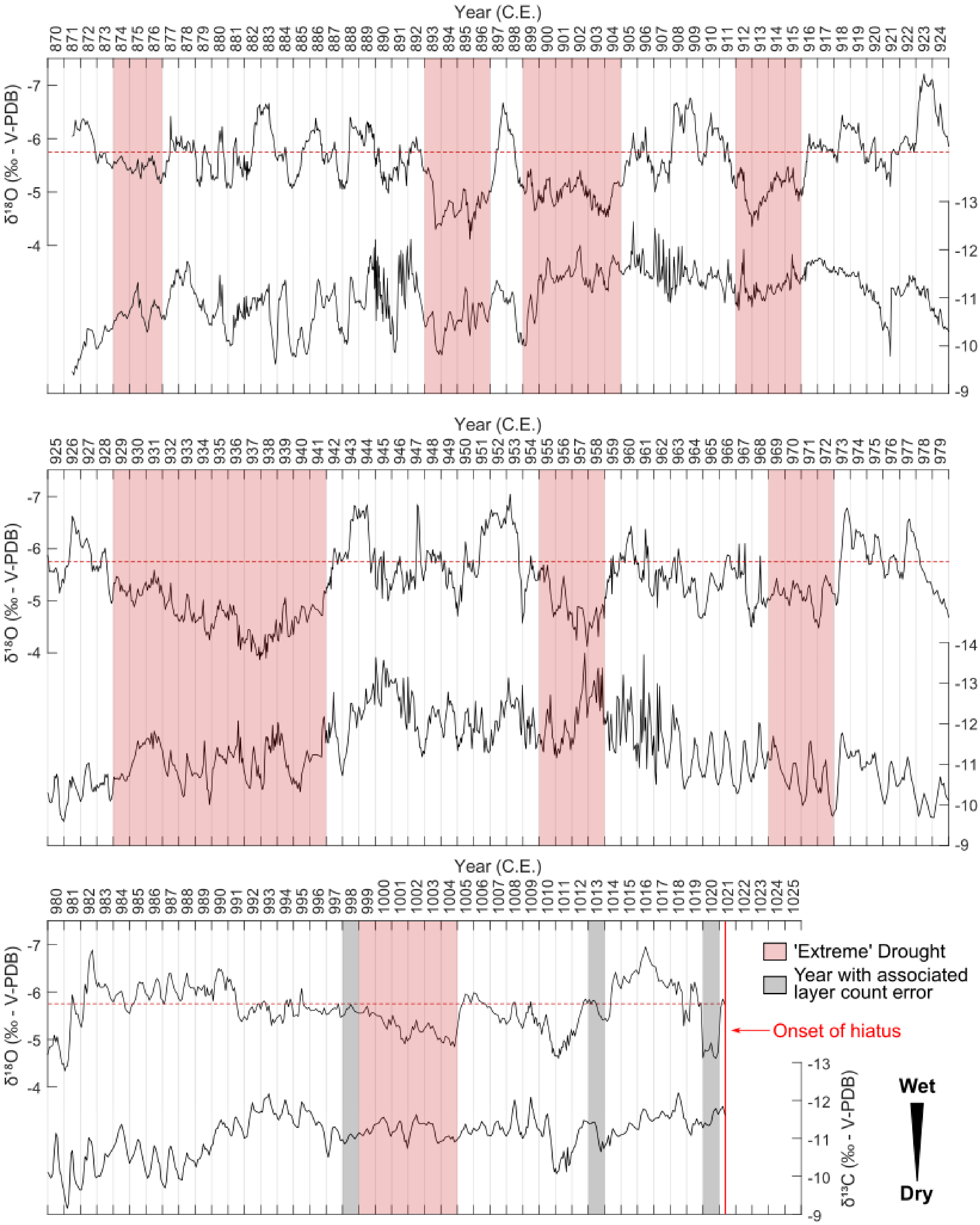

Year (C.E.)
δ¹⁸O (‰ - V-PDB)
δ¹³C (‰ - V-PDB)
'Extreme' Drought
Year with associated layer count error
Onset of hiatus
Wet
Dry

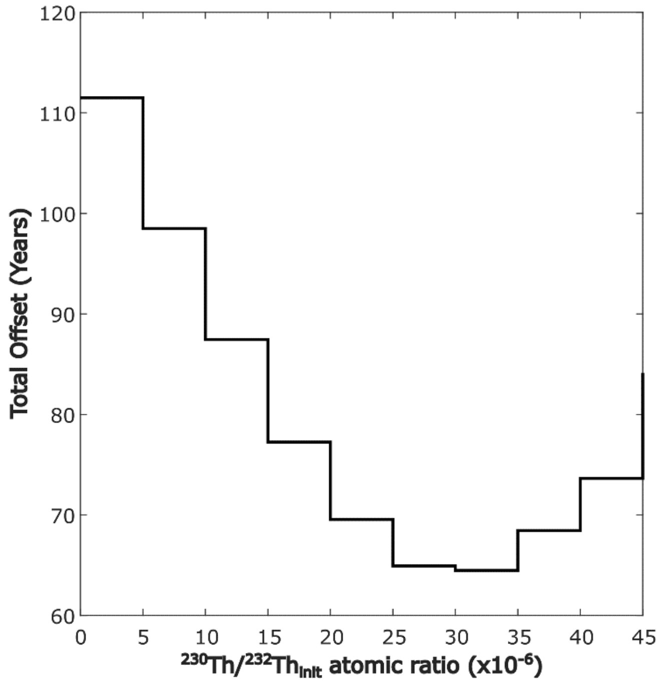


***Fig. S3. Optimisation of Th$_{init}$.*** *Total summed difference between the date-to-date spacing produced by each Th$_{init}$ atomic ratio test, and the known true chronological spacing from the layer count.*

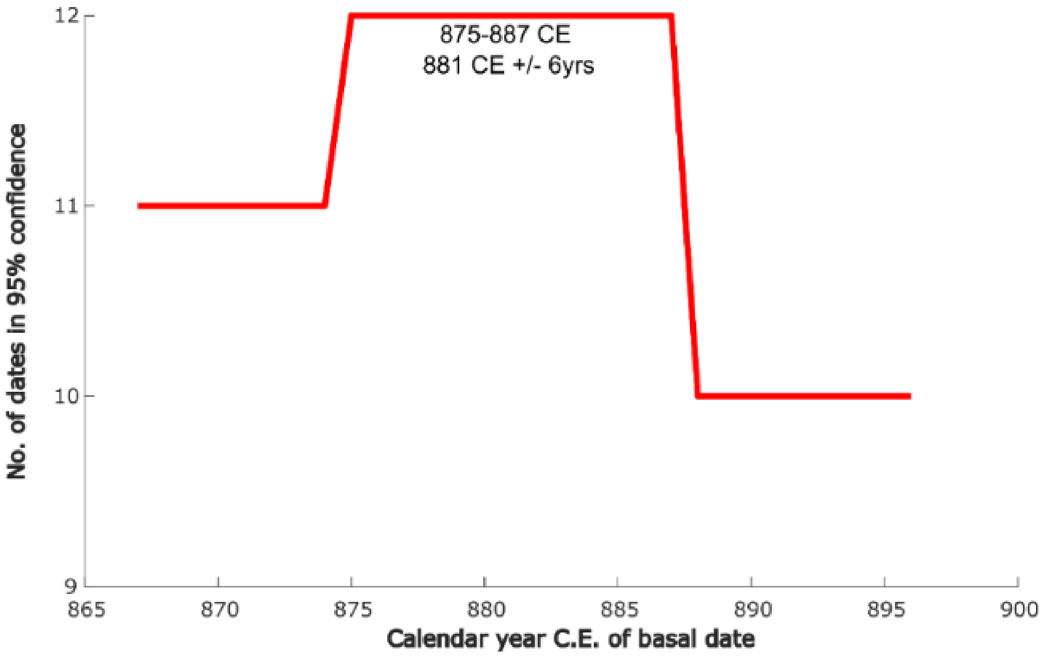


***Fig. S4. Fit test for age model tethers.*** *Results of testing each basal date tether point in the range 865-900 CE with the 12 dates below the depositional hiatus, with the number of 95% confidence intervals passed through in red.*

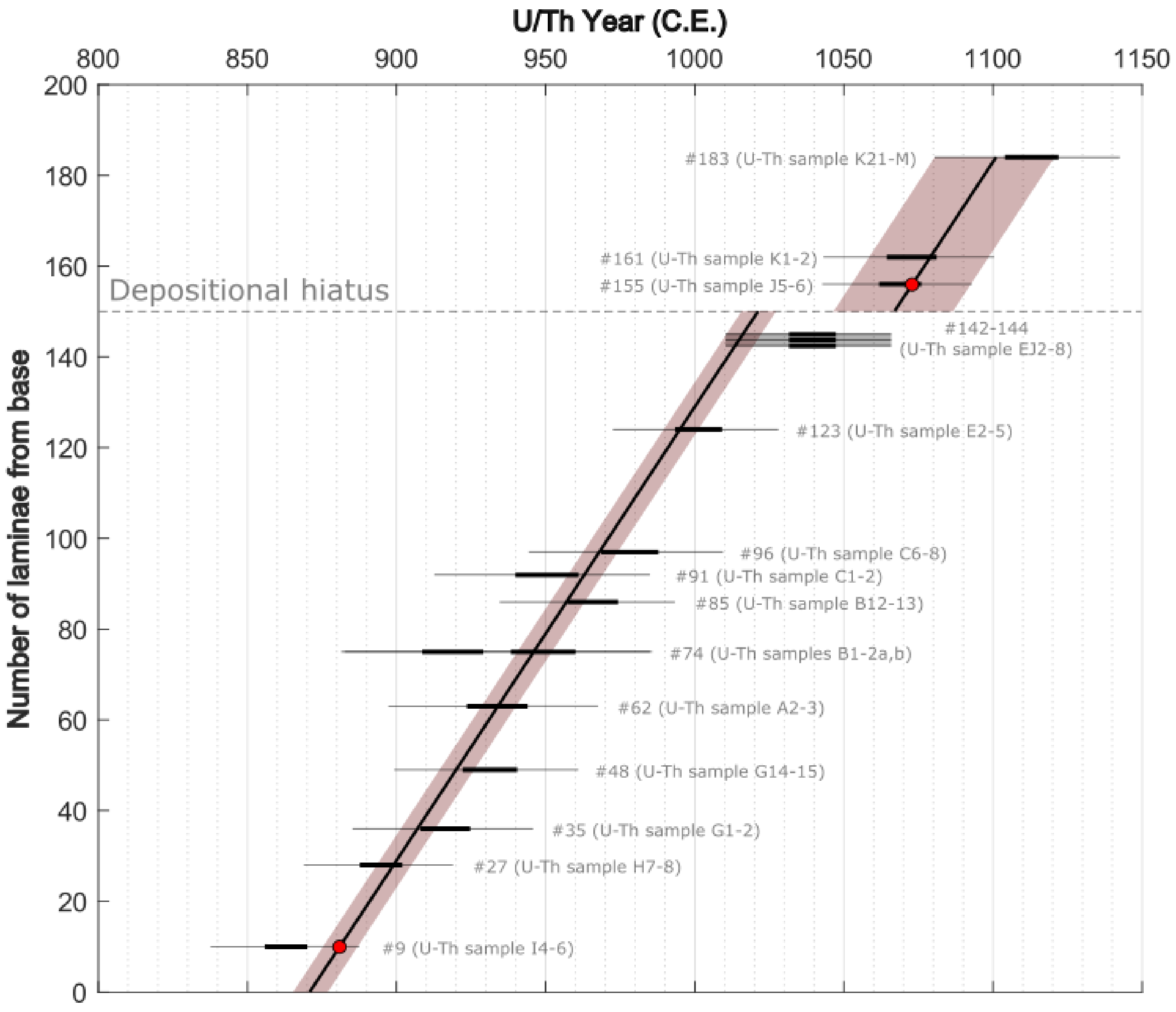


***Fig. S5. Age model.*** *The layer counted chronology tethered to the U-Th dates. Red dots are the chosen tethers of 881 CE (basal) and 1068 CE (above hiatus). The layer count chronology is in black with the confidence interval (CI, as fig. S2) in pale red. Each U-Th date is plotted as a 68 % confidence interval (thick black bar), and 95 % CI (thin grey bars). The depositional hiatus hypothesised by the presence of detrital material is indicated by the dotted grey line. Three laminae (Laminae #126, #142, and #149) have a '1-yr missing' counting error. Two of these three laminae are located between U-Th sample E2-5 at Lamina #123 and U-Th sample EJ2-8 at Lamina #142-144. U-Th sample EJ2-8 is therefore plotted at each of the possible laminae (#142-144) to incorporate uncertainty due to counting error. Note that this is not propagated above the hiatus, as the choice of tether above the horizon of hiatus is independent of that below and relies only on the number of counted laminae above, which does not include any with a '1-yr missing' count error. Replicate U-Th samples were milled and analysed at Lamina #74 (U-Th samples B1-2a and B1-2b).*

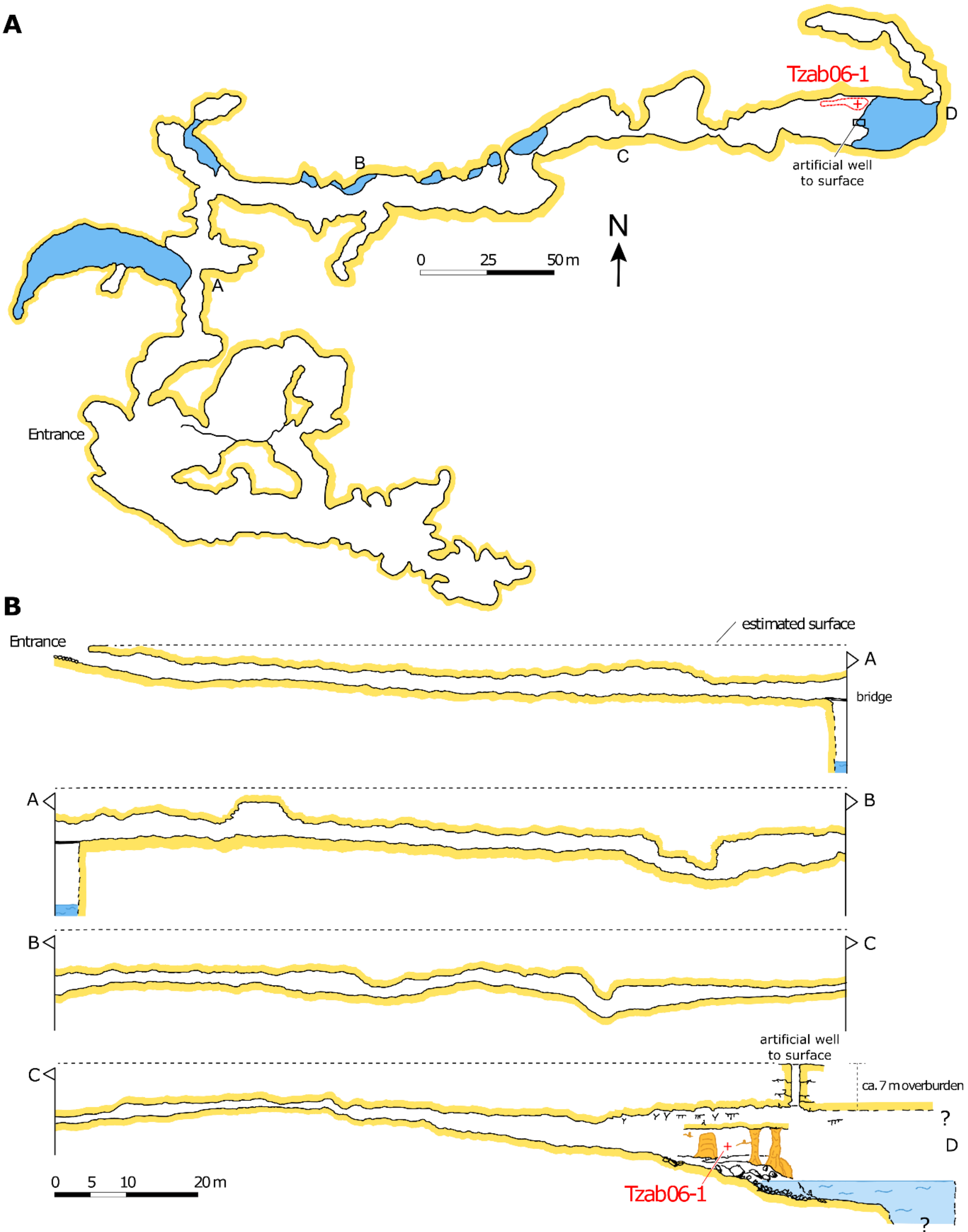


***Fig. S6. Map of Grutas Tzabnah. A.*** *Plan and* ***B.*** *Profile views of Grutas Tzabnah with the Tzab06-1 sample location. The overburden is only ~7 m thick, which ensures rapid transfer of water, and hence environmental signals, from the land surface to the cave. The speleothem sample came from the chamber near the underground lake, 'Cenote 10'.*

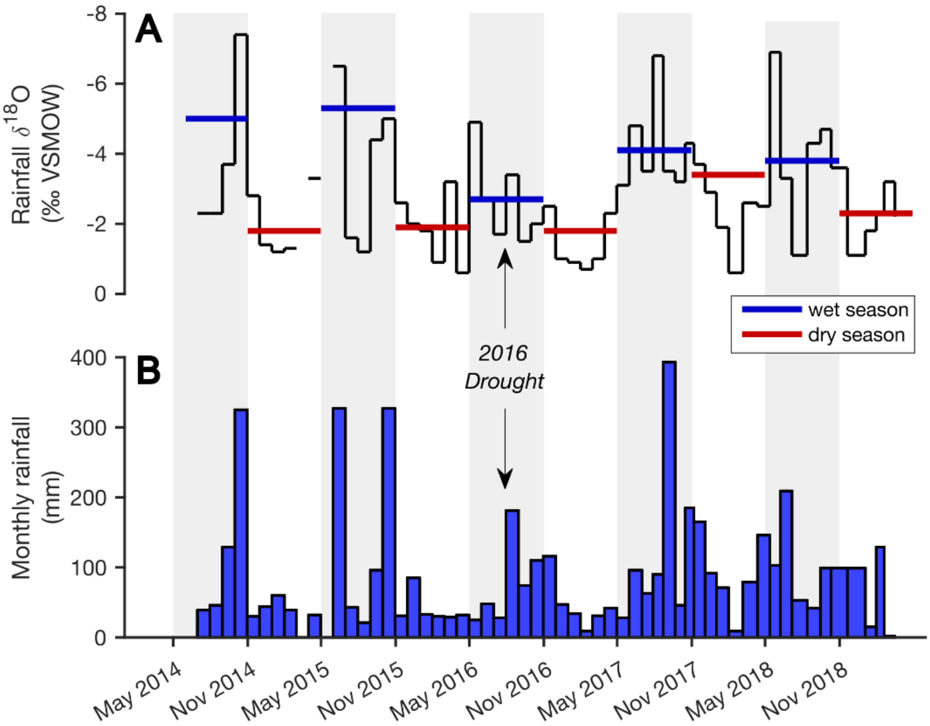


***Fig. S7. Modern drought recorded by $\delta^{18}O_{rw}$ at Río Secreto.*** *Río Secreto cave site (Northern Lowlands, ~250 km east of Grutas Tzabnah) monitored (a) monthly rainfall $\delta^{18}O$ averages and (b) monthly rainfall amount averages, 2014-2019. Data provided in Supplemental Table A of (*36*). Long-term monthly averages are calculated from measured samples of rainwater collected over 30 ± 5 days. Grey vertical bars indicate the generalised wet season, May through October. Horizontal lines in (a) indicate rainwater amount-weighted average rainwater $\delta^{18}O$ values for the wet season (blue) and dry season (red) each year. Note the less negative amount-weighted average rainwater $\delta^{18}O$ values of the wet season during the 2016 drought, compared to other years. All data from (*35,36*).*

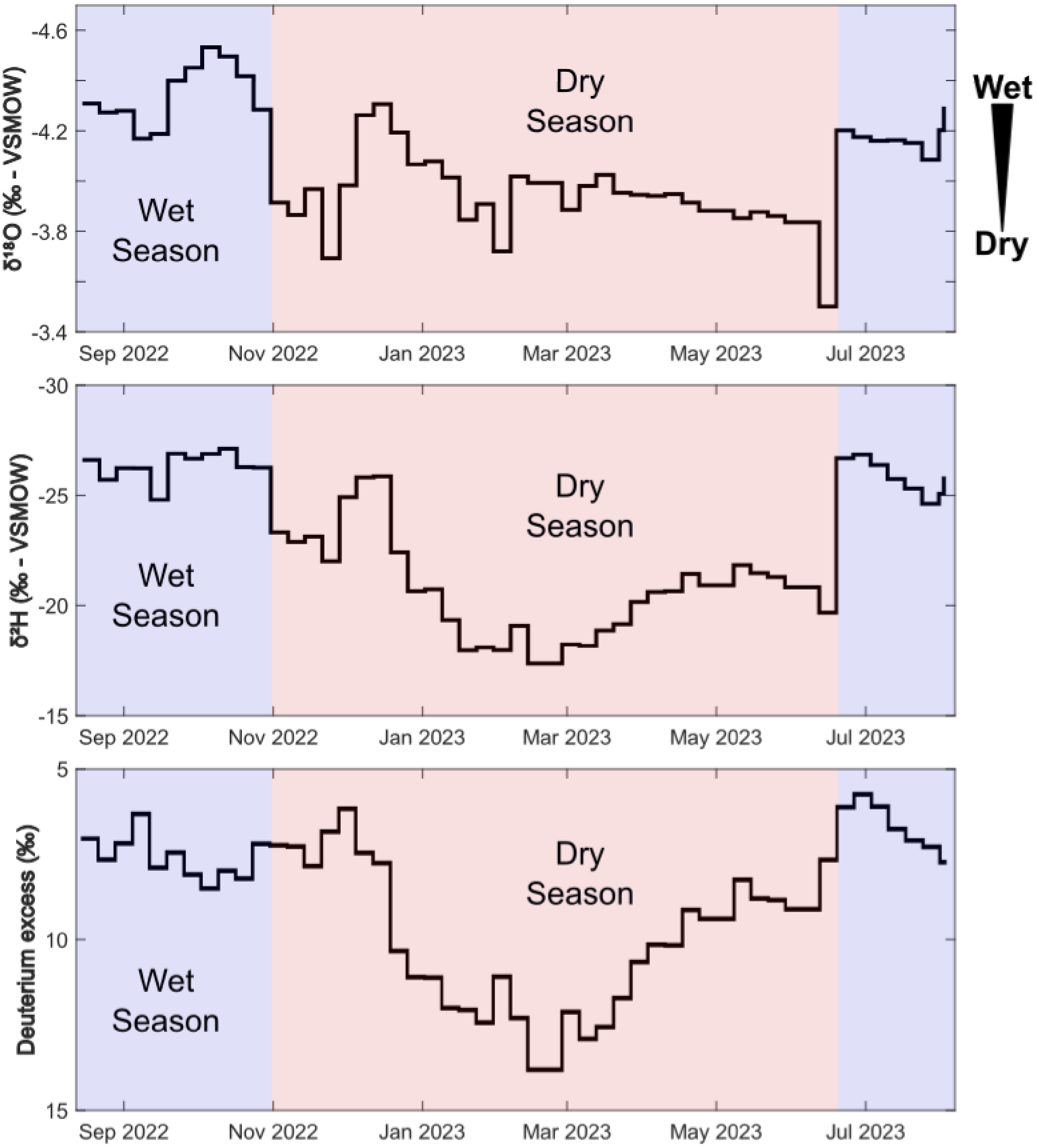


***Fig. S8. Monitored dripwater seasonality at Grutas Tzabnah.*** *$\delta^{18}O_{dw}$, $\delta^{2}H_{dw}$, and deuterium excess measured weekly near the (approximate) location of Tzab06-1 over 1 year from August 13$^{th}$, 2022, to August 8$^{th}$, 2023. Deuterium excess is defined as $\delta^2H - 8*\delta^{18}O$ (*100*). Wet and Dry seasons are here defined by fluctuations in $\delta^{18}O_{dw}$ and $\delta^2H_{dw}$, which emphasise the seasonal variability.*

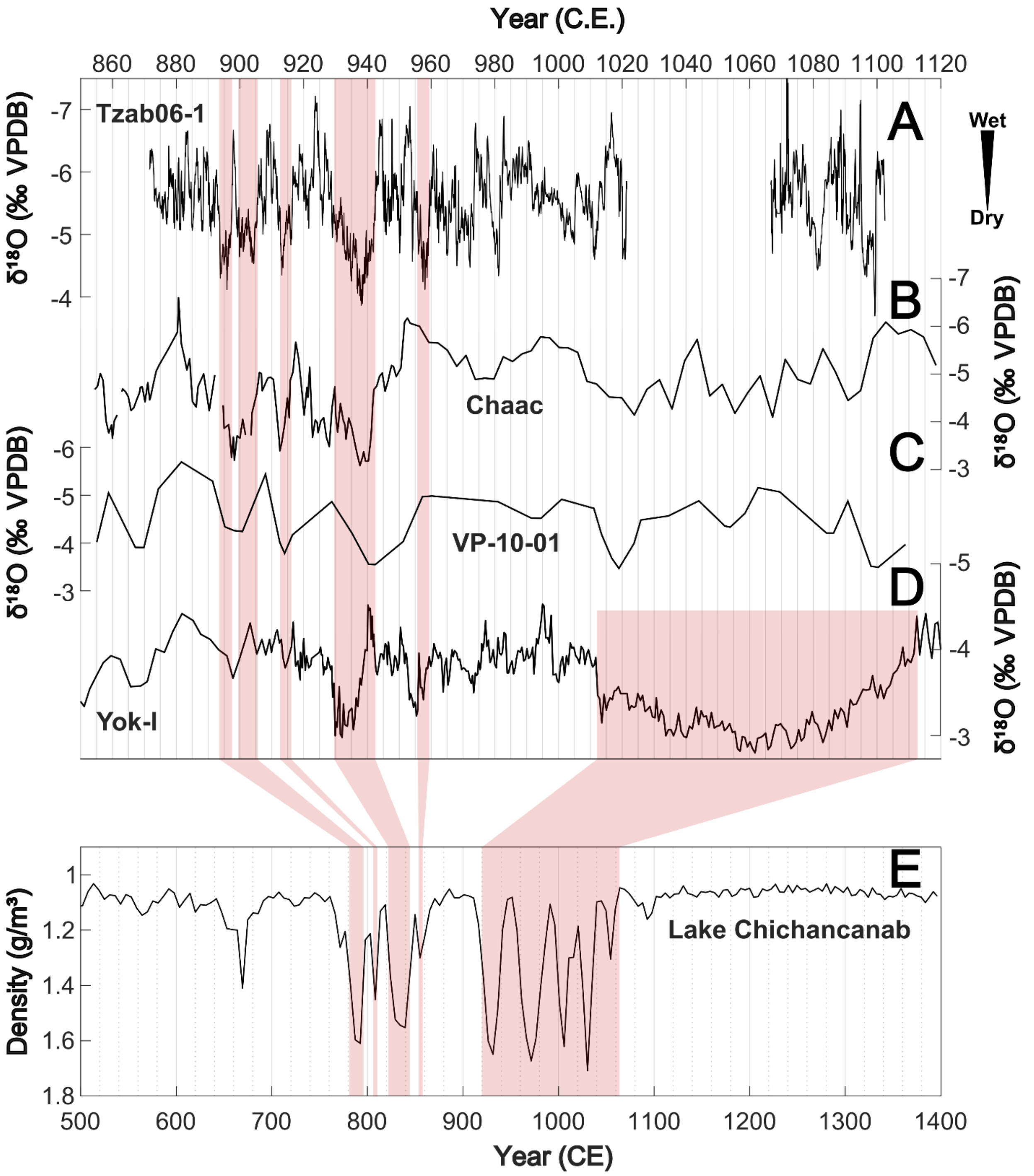


***Fig. S9. Expanded comparison of Tzab06-1 and other regional records. A.*** *Tzab06-1 speleothem,* ***B.*** *the Chaac speleothem from the same cave (*15*),* ***C.*** *the VP-10-01 speleothem (*26,86*), which is tuned to Chaac* ***D.*** *the Yok-I speleothem from southern Belize (*16*), and* ***E.*** *Lake Chichancanab sediment density, indicative of gypsum layers (*13,87*). Red bars are Tzab06-1 droughts of ≥3-yr duration, with the latter indicating the coincidence of the second set of gypsum horizons in Lake Chichancanab with the 'megadrought' in Yok-I.*

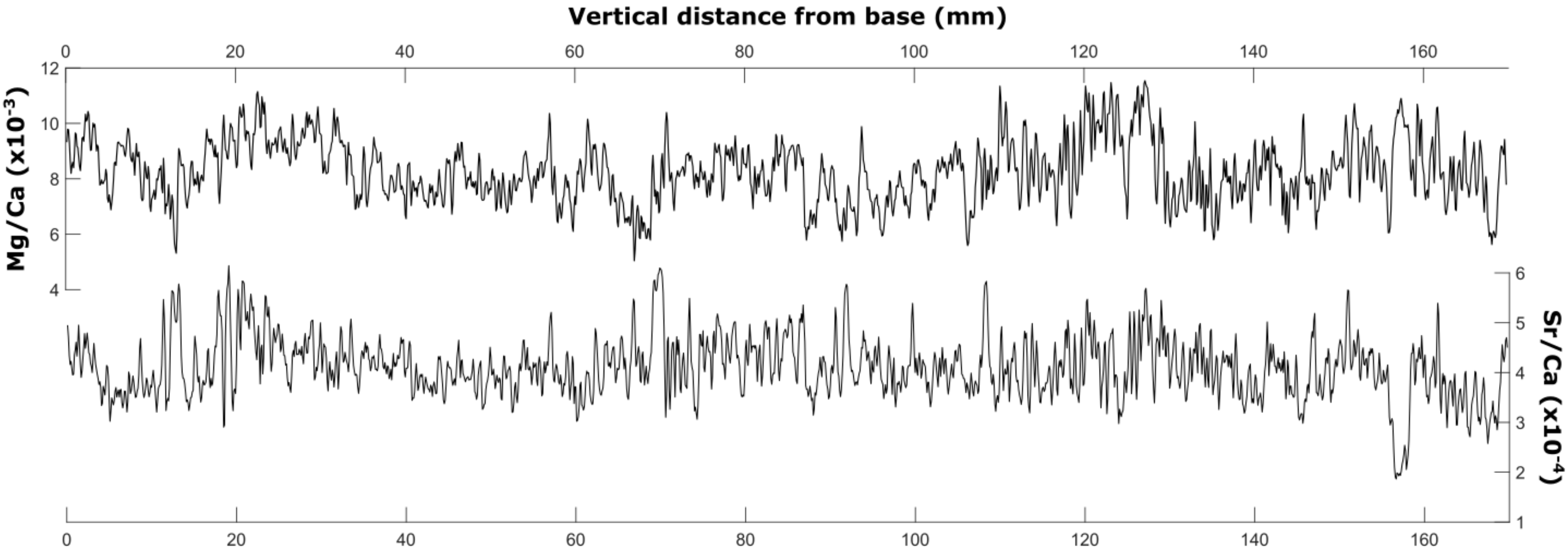


.

***Fig. S10. Tzab06-1 Mg/Ca and Sr/Ca.*** *Mg/Ca (top) and Sr/Ca ratios (bottom) in stalagmite Tzab06-1. Measurements at 10-µm spatial resolution are plotted as a 100-pt moving average (1 mm window) after removal of outliers, then sampled every 100 µm.*

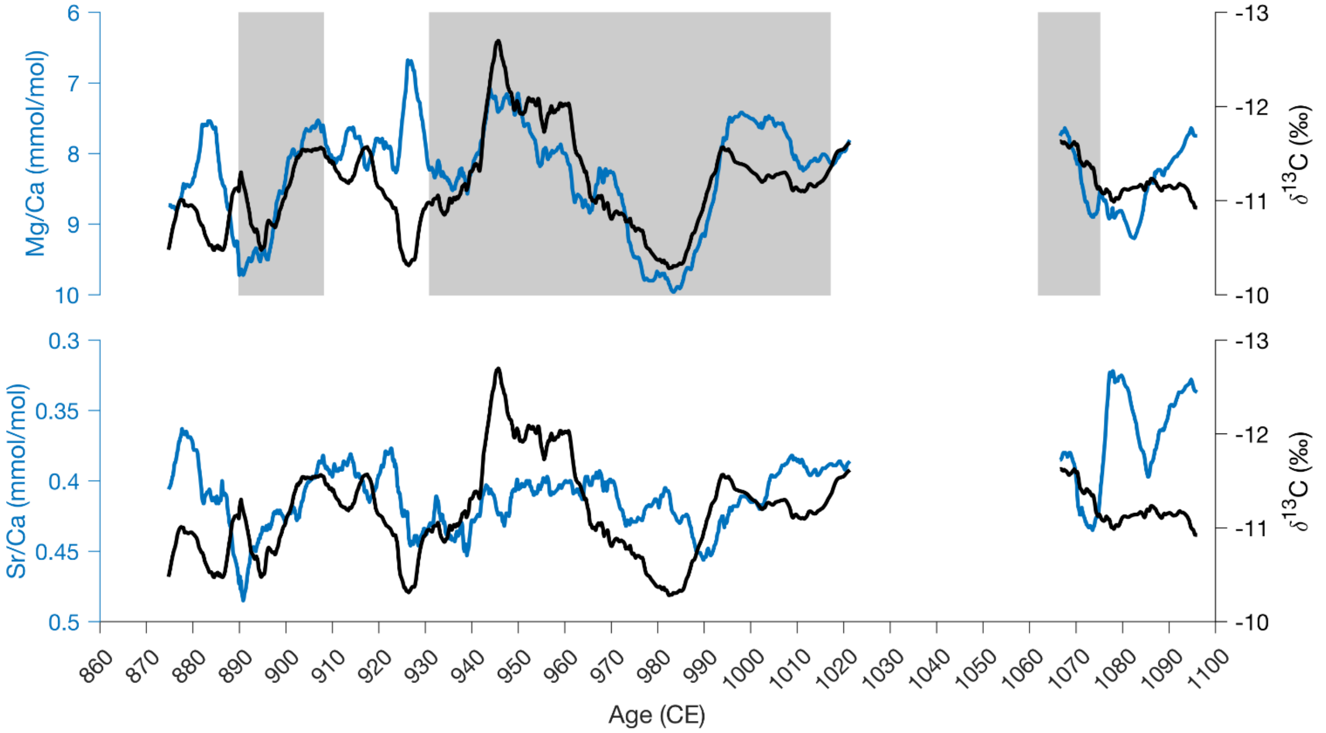


***Fig. S11. Tzab06-1 $\delta^{13}C_{cc}$ and trace elements.*** *Tzab06-1 Mg/Ca, Sr/Ca (both blue) and $\delta^{13}C$ (black). All records were sampled every 100µm, then averaged over a moving 5-mm window. Grey windows indicate where Mg/Ca correlates with $\delta^{13}C$.*

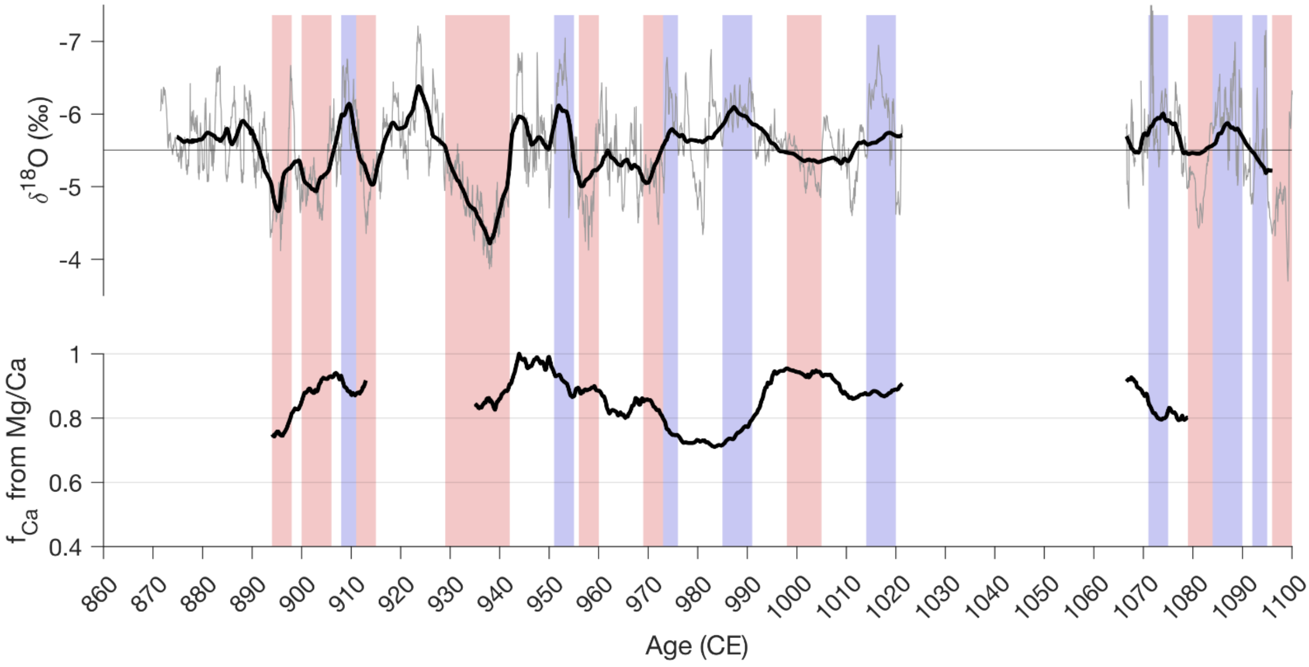


***Fig. S12. Tzab06-1 PCP reconstruction.*** *Tzab06-1 $\delta^{18}O$ (with 5-mm running average) and estimated approximate $f_{Ca}$ from stalagmite Mg/Ca. $f_{Ca}$ = 1 indicates no PCP. $f_{Ca}$ < 1 indicates some effect of PCP. Red and blue bars are identified droughts and wet periods as per fig. S12.*

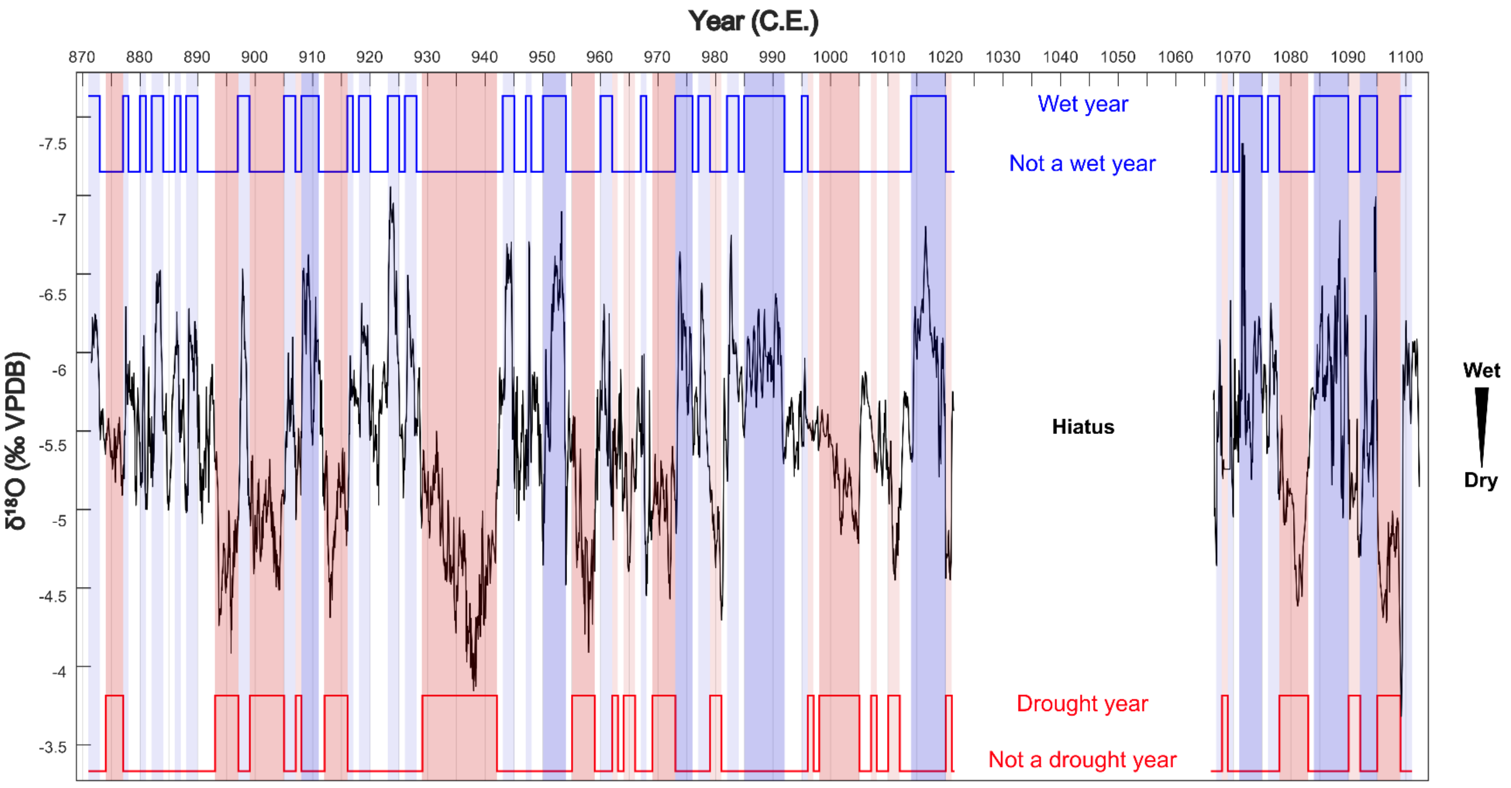


***Fig. S13. Assignment of droughts and wet periods.*** *Droughts (red) and wet periods (blue) as defined herein. Three or more consecutive years of drought/high rainfall are classed as extreme droughts/wet events (sensu 44) and are highlighted in darker red/blue. The red and blue solid lines plot whether each given year met the threshold for a drought or wet year.*

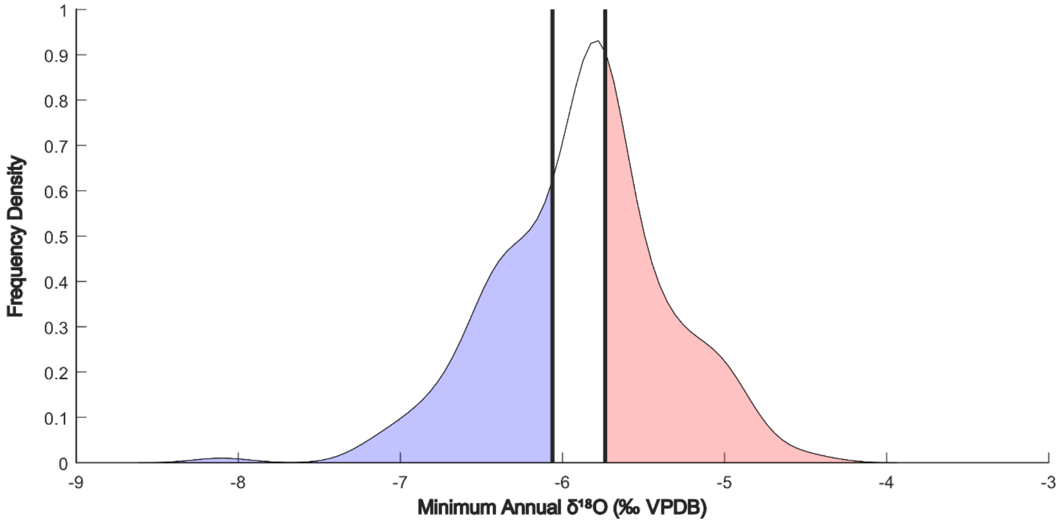


***Fig. S14. Drought and wet period assignment thresholds.*** *Distribution of annual minimum $\delta^{18}O_{cc}$ values, with the thresholds for drought and wet years, $1\sigma$ from the mean in each case, plotted as black vertical lines.*

# Supplementary Tables

Supplementary Tables S1-4 are presented in the accompanying spreadsheet.

***Table S1. Stable Oxygen and Carbon isotope ratios in Tzab06-1.*** *$\delta^{18}O$ and $\delta^{13}C$ results for Tzab06-1, against sample number, vertical distance up the specimen from the base, lamina count, and modelled age.*

***Table S2. Water isotopic composition results from Grutas Tzabnah.*** *Water isotopic $\delta^{18}O$ and $\delta^{2}H$ data from dripwater samples collected weekly at the (approximate) site of Tzab06-1 from August 2022 to August 2023.*

***Table S3. Trace element data for Tzabo6-1.*** *Magnesium and Strontium to Calcium ratios in Tzab06-1, against vertical distance up the specimen from the base.*

***Table S4. U-Th results and age model for Tzab06-1.*** *Uranium and Thorium concentrations and isotopic compositions for stalagmite Tzab06-1 with the calculated disequilibrium ages and final age model.*

| Puuc Region | | | Chichén Itzá | | |
|---|---|---|---|---|---|
| **Site Name** | **Long Count Date** | **Gregorian Year (CE)** | **Site Name** | **Long Count** | **Gregorian Year (CE)** |
| **Itzimte** | 10.01.00.00.00 | 849 | Chichén Itzá | 10.01.15.03.06.? | 865 |
| **Oxkintok** | 10.01.00.00.00 | 849 | Chichén Itzá | 10.02.00.00.00 | 869 |
| **Oxkintok** | 10.01.10.00.00? | 859 | Chichén Itzá | 10.02.00.00.00 | 869 |
| **Oxkintok** | 10.01.10.00.00? | 859 | Chichén Itzá | 10.02.01.00.00 | 870 |
| **Oxkintok** | 10.01.10.00.00? | 859 | Chichén Itzá | 10.02.02.00.00 | 871 |
| **Kabah** | 10.01.10.00.11 | 859 | Chichén Itzá | 10.02.09.01.09 | 878 |
| **Kabah** | 10.01.10.00.11 | 860 | Chichén Itzá | 10.02.09.00.00 | 878 |
| **Labna** | 10.01.13.00.00 | 862 | Chichén Itzá | 10.02.10.11.07 | 879 |
| **Sayil** | 10.03.00.00.00 | 889 | Chichén Itzá | 10.02.10.00.00 | 879 |
| **Sayil** | 10.03.00.00.00 | 889 | Chichén Itzá | 10.02.10.00.00 | 879 |
| **Uxmal** | 10.03.06.00.00 | 895 | Chichén Itzá | 10.02.12.02.04 | 881 |
| **Uxmal** | 10.03.11.15.14 | 900 | Chichén Itzá | 10.02.12.01.08 | 881 |
| **Uxmal** | 10.03.15.16.14 | 904 | Chichén Itzá | 10.02.12.02.04 | 881 |
| **Uxmal** | 10.03.17.12.01 | 906 | Chichén Itzá | 10.02.12.01.08 | 881 |
| **Uxmal** | 10.03.18.09.12 | 907 | Chichén Itzá | 10.02.12.01.09? | 881 |
| **Itzimte** | 10.04.01.00.00 | 910 | Chichén Itzá | 10.02.13.13.01 | 882 |
| **Pixoy** | 09.14.00.00.00 | 910 | Chichén Itzá | 10.02.13.13.01 | 882 |
| | | | Chichén Itzá | 10.03.00.00.00 | 889 |
| | | | Chichén Itzá | 10.03.00.00.00 | 889 |
| | | | Chichén Itzá | - | 994 |
| | | | Chichén Itzá | 10.08.10.11.00 | 998 |
| | | | Chichén Itzá | 10.??.08.??.?? | 998 |

***Table S5. Puuc Long Count Dates 840-1000 CE.*** *Selected Long Count dates from the compilation of Hoggarth et al., (5), as used in Fig. 4 (Main text). See original reference for discussion of the Chichén Itzá dates 994-998 CE.*

## Example Analysis Script

The following script was used as part of data analysis in this study, originally run in Matlab R2023a. The script is not required to replicate the data, and alternative software could be used as appropriate. All data are provided in the Supplementary Tables accompanying this article.

Example script for age calculation from U/Th

```
Excelinput = readtable('Table4.xlsx');
IsoRatiosAgeEqn_data = table2array(Excelinput);
date_measured = IsoRatiosAgeEqn_data(:,15); % date analysed on the MC-ICP-MS, CE.
U234_U238 = IsoRatiosAgeEqn_data(:,9); % 234U/238U ratio
Th230_U238 = IsoRatiosAgeEqn_data(:,11); % 230Th/238U ratio
Th232_U238 = IsoRatiosAgeEqn_data(:,13); % 232Th/238U ratio
U234_U238_1s = IsoRatiosAgeEqn_data(:,10); % 1σ for 234U/238U ratio
Th230_U238_1s = IsoRatiosAgeEqn_data(:,12); % 1σ for 230Th/238U ratio
Th232_U238_1s = IsoRatiosAgeEqn_data(:,14); % 1σ for 232Th/238U ratio

% initial 230Th/232Th atom ratio, calculated in Methods.
lowbound = 25e-6; % lower bound for initial 230Th/232Th atom ratio
upbound = 35e-6; % upper bound for initial 230Th/232Th atom ratio
Agestart = 1000; % starting value for fzero
n = 10000;
Lam238 = 1.55125E-10; %Cheng et al. 2013
Lam234 = 2.82206E-06; %Cheng et al. 2013
Lam230 = 9.1705E-06; %Cheng et al. 2013
Lam232 = 4.9475E-11; %Cheng et al. 2013
Th230_Th232_init_mc = unifrnd(lowbound,upbound,[n 1]).*(Lam230/Lam232);

U234_U238_mc = [];
Th230_U238_mc = [];
Th232_U238_mc = [];
[m,~] = size(IsoRatiosAgeEqn_data);
for i = 1:m
    U234_U238_mc(i,:) = U234_U238(i) + U234_U238_1s(i).*randn(1,n);
    Th230_U238_mc(i,:) = Th230_U238(i) + Th230_U238_1s(i).*randn(1,n);
    Th232_U238_mc(i,:) = Th232_U238(i) + Th232_U238_1s(i).*randn(1,n);
end

Age_uncorr = [];
Age_corr = [];
U234_U238_init = [];
for i = 1:m
    for j = 1:n
        Age_uncorr(i,j) = fzero(@(t) ...
            Th230_U238_mc(i,j)-(1-exp(-Lam230*t))-...
            Lam230/(Lam230-Lam234)*(exp(-Lam234*t)-exp(-Lam230*t))*exp(Lam234*t)*
                           (U234_U238_mc(i,j)-1),Agestart);
        Age_corr(i,j) = fzero(@(t) ...
            Th230_U238_mc(i,j)-(1-exp(-Lam230*t))-...
            exp(-Lam230*t)*Th230_Th232_init_mc(j)*Th232_U238_mc(i,j)-...
            Lam230/(Lam230-Lam234)*(exp(-Lam234*t)-exp(-Lam230*t))*exp(Lam234*t)*
                           (U234_U238_mc(i,j)-1),Agestart);
        U234_U238_init(i,j) = U234_U238_mc(i,j)*exp(Lam234*Age_corr(i,j));
    end
end
```

```
% outputs, all in years CE: uncorrected mean age, 95% interval lower, 95% interval
upper, corrected mean age, 95% interval lower, 95% interval upper, Initial 234U/238U
ratio, 95% interval lower, 95% interval upper
ages_final_CE = [];
for i = 1:m
    ages_final_CE(i,:) = [date_measured(i)-mean(Age_uncorr(i,:)) ...
        mean(Age_uncorr(i,:))-prctile(Age_uncorr(i,:),5) ...
        prctile(Age_uncorr(i,:),95)-mean(Age_uncorr(i,:)) ...
        date_measured(i)-mean(Age_corr(i,:)) ...
        mean(Age_corr(i,:))-prctile(Age_corr(i,:),5) ...
        prctile(Age_corr(i,:),95)-mean(Age_corr(i,:)) ...
        mean(U234_U238_init(i,:)) ...
        mean(U234_U238_init(i,:))-prctile(U234_U238_init(i,:),5) ...
        prctile(U234_U238_init(i,:),95)-mean(U234_U238_init(i,:))];
end
```

**REFERENCES AND NOTES**

1. T. P. Culbert, *The Classic Maya Collapse* (University of New Mexico Press, 1973).

2. C. E. Ebert, K. M. Prufer, M. J. Macri, B. Winterhalder, D. J. Kennett, Terminal long count dates and the disintegration of classic period Maya polities. *Anc. Mesoam.* **25**, 337–356 (2015).

3. K. M. Prufer, A. E. Thompson, A. D. Wickert, D. J. Kennett, The development and disintegration of a Classic Maya center and its climate context. *Prog. Phys. Geogr.* **47**, 205–226 (2023).

4. D. Graña-Behrens, C. Prager, E. Wagner, The hieroglyphic inscription of the High Priest's Grave at Chichén Itzá, Yucatán, Mexico. *Mexicon* **21**, 61–66 (1999).

5. J. A. Hoggarth, S. F. M. Breitenbach, B. J. Culleton, C. E. Ebert, M. A. Masson, D. J. Kennett, The political collapse of Chichén Itzá in climatic and cultural context. *Glob. Planet. Change* **138**, 25–42 (2016).

6. B. H. Dahlin, Climate and prehistory on the Yucatan peninsula. *Clim. Change* **5**, 245–263 (1990).

7. R. B. Gill, *The Great Maya Droughts: Water, Life, and Death* (University of New Mexico Press, 2000).

8. D. Webster, *The Fall of the Ancient Maya: Solving the Mystery of the Maya Collapse* (Thames & Hudson Ltd., 2002).

9. A. Demarest, *Ancient Maya: The Rise and Fall of a Rainforest Civilization* (Cambridge Univ. Press, 2004), vol. 3.

10. J. J. Aimers, What Maya collapse? Terminal classic variation in the Maya Lowlands. *J. Archaeol. Res.* **15**, 329–377 (2007).

11. B. L. Turner, J. A. Sabloff, Classic period collapse of the Central Maya Lowlands: Insights about human–environment relationships for sustainability. *Proc. Natl. Acad. Sci. U.S.A.* **109**, 13908–13914 (2012).

12. D. A. Hodell, J. H. Curtis, M. Brenner, Possible role of climate in the collapse of Classic Maya civilization. *Nature* **375**, 391–394 (1995).

13. D. A. Hodell, M. Brenner, J. H. Curtis, Terminal Classic drought in the northern Maya Lowlands inferred from multiple sediment cores in Lake Chichancanab (Mexico). *Quat. Sci. Rev.* **24**, 1413–1427 (2005).

14. G. H. Haug, D. Günther, L. C. Peterson, D. M. Sigman, K. A. Hughen, B. Aeschlimann, Climate and the collapse of Maya civilization. *Science* **299**, 1731–1735 (2003).

15. M. Medina-Elizalde, S. J. Burns, D. W. Lea, Y. Asmerom, L. von Gunten, V. J. Polyak, M. Vuille, A. Karmalkar, High resolution stalagmite climate record from the Yucatán Peninsula spanning the Maya terminal classic period. *Earth Planet. Sci. Lett.* **298**, 255–262 (2010).

16. D. J. Kennett, S. F. M. Breitenbach, V. V. Aquino, Y. Asmerom, J. Awe, J. U. L. Baldini, P. Bartlein, B. J. Culleton, C. E. Ebert, C. Jazwa, M. J. Macri, N. Marwan, V. J. Polyak, K. M. Prufer, H. E. Ridley, H. Sodemann, B. Winterhalder, G. H. Haug, Development and disintegration of Maya political systems in response to climate change. *Science* **338**, 788–791 (2012).

17. Y. Asmerom, J. U. L. Baldini, K. M. Prufer, V. J. Polyak, H. E. Ridley, V. V. Aquino, L. M. Baldini, S. F. M. Breitenbach, D. J. Kennett, Intertropical convergence zone variability in the Neotropics during the Common Era. *Sci. Adv.* **6**, eaax3644 (2020).

18. D. A. Wilhite, M. H. Glantz, Understanding the drought phenomenon: The role of definitions. *Water Int.* **10**, 111–120 (1985).

19. G. Iannone, Ed., *The Great Maya Droughts in Cultural Context: Case Studies in Resilience and Vulnerability* (University Press of Colorado, 2014).

20. J. Haldon, L. Mordechai, T. P. Newfield, A. F. Chase, A. Izdebski, P. Guzowski, I. Labuhn, N. Roberts, History meets palaeoscience: Consilience and collaboration in studying past societal responses to environmental change. *Proc. Natl. Acad. Sci. U.S.A.* **115**, 3210–3218 (2018).

21. C. Isendahl, N. P. Dunning, J. A. Sabloff, 4 Growth and decline in Classic Maya Puuc political economies. *Archeol. Pap. Am. Anthropol. Assoc.* **24**, 43–55 (2014).

22. J. Yaeger, D. A. Hodell, "The collapse of Maya civilization: Assessing the interaction of culture, climate, and environment," in *El Niño, Catastrophism, and Culture Change in Ancient America*, D. H. Sandweiss, J. Quilter, Eds. (Dumbarton Oaks Research Library and Collection, 2008), pp. 187–242.

23. B. Mendoza, V. García-Acosta, V. Velasco, E. Jáuregui, R. Díaz-Sandoval, Frequency and duration of historical droughts from the 16th to the 19th centuries in the Mexican Maya lands, Yucatan Peninsula. *Clim. Change* **83**, 151–168 (2007).

24. P. M. Douglas, M. Pagani, M. A. Canuto, M. Brenner, D. A. Hodell, T. I. Eglington, J. H. Curtis, Drought, agricultural adaptation, and sociopolitical collapse in the Maya Lowlands. *Proc. Natl. Acad. Sci. U.S.A.* **112**, 5607–5612 (2015).

25. P. D. Akers, G. A. Brook, L. B. Railsback, F. Liang, G. Ianonne, J. W. Webster, P. P. Reeder, H. Cheng, R. L. Edwards, An extended and higher-resolution record of climate and land use from stalagmite MC01 from Macal Chasm, Belize, revealing connections between major dry events, overall climate variability, and Maya sociopolitical changes. *Palaeogeogr. Palaeoclimatol. Palaeoecol.* **459**, 268–288 (2016).

26. M. P. Smyth, N. P. Dunning, E. M. Weaver, P. van Beynen, D. Ortegón Zapata, "An enigmatic Maya center: Climate change, settlement systems, and water adaptations at Xcoch, Puuc region, Yucatán," in *Recent Investigations in the Puuc Region of Yucatán, Archaeopress Precolumbian Archaeology 8*, M. Rubenstein, Ed., (Archaeopress, 2017), pp. 3–24.

27. H. E. Ridley, Y. Asmerom, J. U. L. Baldini, S. F. M. Breitenbach, V. V. Aquino, K. M. Prufer, B. J. Culleton, V. J. Polyak, F. A. Lechleitner, D. J. Kennett, M. Zhang, N. Marwan, C. G. Macpherson, L. M. Baldini, T. Xiao, J. L. Peterkin, J. Awe, G. H. Haug, Aerosol forcing of the position of the intertropical convergence zone since AD 1550. *Nat. Geosci.* **8**, 195–200 (2015).

28. T. Braun, S. F. M. Breitenbach, V. Skiba, F. A. Lechleitner, E. E. Ray, L. M. Baldini, V. J. Polyak, J. U. L. Baldini, D. J. Kennett, K. M. Prufer, N. Marwan, Decline in seasonal predictability potentially destabilized Classic Maya societies. *Commun. Earth Environ.* **4**, 82 (2023).

29. B. De la Barreda, S. E. Metcalfe, D. S. Boyd, Precipitation regionalization, anomalies and drought occurrence in the Yucatan Peninsula, Mexico. *Int. J. Climatol.* **40**, 4541–4555 (2020)

30. A. Baker, C. L. Smith, C. Jex, I. J. Fairchild, D. Genty, L. Fuller, Annually laminated speleothems: A review. *Int. J. Speleol.* **37**, 193–206 (2008).

31. A. Baker, G. Mariethoz, L. Comas-Bru, A. Hartmann, S. Frisia, A. Borsato, P. C. Treble, A. Asrat, The properties of annually laminated stalagmites-A global synthesis. *Rev. Geophys.* **59**, e2020RG000722 (2021).

32. D. Domínguez-Villar, A. Baker, I. J. Fairchild, R. L. Edwards, A method to anchor floating chronologies in annually laminated speleothems with U–Th dates. *Quat. Geochronol.* **14**, 57–66 (2012).

33. A. Giannini, Y. Kushnir, M. A. Cane, Interannual variability of Caribbean rainfall, ENSO, and the Atlantic Ocean. *J. Climate* **13**, 297–311 (2000).

34. A. M. Mestas-Nuñez, D. B. Enfield, C. Zhang, Water vapor fluxes over the Intra-Americas Sea: Seasonal and interannual variability and associations with rainfall. *J. Climate* **20**, 1910–1922 (2007).

35. F. Lases-Hernandez, M. Medina-Elizalde, S. Burns, M. DeCesare, Long-term monitoring of drip water and groundwater stable isotopic variability in the Yucatán Peninsula: Implications for recharge and speleothem rainfall reconstruction. *Geochim. Cosmochim. Acta* **246**, 41–59 (2019).

36. F. Lases-Hernández, M. Medina-Elizalde, A. B. Frappier, Drip water $\delta^{18}O$ variability in the northeastern Yucatán Peninsula, Mexico: Implications for tropical cyclone detection and rainfall reconstruction from speleothems. *Geochim. Cosmochim. Acta* **285**, 237–256 (2020).

37. S. A. Ellis, K. M. Cobb, J. W. Moerman, J. W. Partin, A. Landry Bennett, J. Malang, H. Gerstner, A. A. Tuen, Extended cave drip water time series captures the 2015–2016 El Niño in northern Borneo. *Geophys. Res. Lett.* **47**, e2019GL086363 (2020).

38. J. U. L. Baldini, F. A. Lechleitner, S. F. M. Breitenbach, J. van Hunen, L. M. Baldini, P. M. Wynn, R. A. Jamieson, H. E. Ridley, A. J. Baker, I. W. Walczak, J. Fohlmeister, Detecting and quantifying palaeoseasonality in stalagmites using geochemical and modelling approaches. *Quat. Sci. Rev.* **254**, 106784 (2021).

39. M. Medina-Elizalde, E. J. Rohling, Collapse of Classic Maya civilization related to modest reduction in precipitation. *Science* **335**, 956–959 (2012).

40. S. L. Fedick, *The Managed Mosaic: Ancient Maya Agriculture and Resource Use* (University of Utah Press, 1996).

41. G. A. Islebe, N. Torrescano-Valle, M. Valdez-Hernández, A. Carrillo-Bastos, A. A. Aragón-Moreno, Maize and ancient Maya droughts. *Sci. Rep.* **12**, 22272 (2022).

42. N. P. Dunning, T. P. Beach, S. Luzzadder-Beach, Kax and kol: Collapse and resilience in lowland Maya civilization. *Proc. Natl. Acad. Sci. U.S.A.* **109**, 3652–3657 (2012).

43. L. Kuil, G. Carr, A. Viglione, A. Prskawetz, G. Blöschl, Conceptualizing socio-hydrological drought processes: The case of the Maya collapse. *Water Resour. Res.* **52**, 6222–6242 (2016)

44. S. L. Fedick, L. S. Santiago, Large variation in availability of Maya food plant sources during ancient droughts. *Proc. Natl. Acad. Sci. U.S.A.* **119**, e2115657118 (2022).

45. G. Horseman, S. Morrell-Hart, C. Golden, A. Scherer, Sustainability models of ancient Maya agriculture in the upper Usumacinta River basin of Mexico and Guatemala. *Lat. Am. Antiq.* **35**, 946–964 (2024).

46. N. P. Dunning, T. Beach, E. Graham, D. Lentz, S. Luzzadder-Beach, "Maize, manioc, mamay, and more: Pre-Columbian lowland Maya agriculture," in *The Archaeology of Caribbean and*

*Circum-Caribbean Farmers (5000 BC – AD1500)*, B. Reid, Ed., (Routledge, 2018), pp. 329–352.

47. V. G. Acosta, J. M. P. Zevallos, A. M. del Villar, *Desastres agrícolas en México: Epocas prehispánica y colonial (958–1822)* (CIESAS, 2003), vol. 1.

48. V. G. Acosta, J. M. P. Zevallos, A. M. del Villar, *Desastres agrícolas en México: Catálogo histórico (1822–1900)* (CIESAS, 2004), vol. 2.

49. L. Tan, W. Liu, T. Wang, P. Cheng, J. Zang, X. Wang, L. Ma, D. Li, J. Lan, R. L. Edwards, H. Cheng, H. Xu, L. Ai, Y. Gao, Y. Cai, A multiple-proxy stalagmite record reveals historical deforestation in central Shandong, northern China. *Sci. China Earth Sci.* **63**, 1622–1632 (2020).

50. J. A. Hoggarth, M. Restall, J. W. Wood, D. J. Kennett, Drought and its demographic effects in the Maya Lowlands. *Curr. Anthropol.* **58**, 82–113 (2017).

51. N. P. Dunning, J. K. Kowalski, Lords of the hills: Classic Maya settlement patterns and political iconography in the Puuc region, Mexico. *Anc. Mesoam.* **5**, 63–95 (1994).

52. J. K. Kowalski, N. P. Dunning, "The architecture of Uxmal: The symbolics of statemaking at a Puuc Maya regional capital," in *Mesoamerican Architecture as a Cultural Symbol*, J. K. Kowalski, Ed., (Oxford Univ. Press, 1999), pp. 273–297.

53. D. J. Kennett, M. A. Masson, C. Peraza Lope, S. Serafin, R. J. George, T. C. Spencer, J. A. Hoggarth, B. J. Culleton, T. K. Harper, K. M. Prufer, S. Milbrath, B. W. Russell, E. Uc González, W. C. McCool, V. V. Aquino, E. H. Paris, J. H. Curtis, N. Marwan, M. Zhang, Y. Asmerom, V. J. Polyak, S. A. Carolin, D. H. James, A. J. Mason, G. M. Henderson, M. Brenner, J. U. L. Baldini, S. F. M. Breitenbach, D. A. Hodell, Drought-induced civil conflict among the ancient Maya. *Nat. Commun.* **13**, 3911 (2022).

54. L. J. Lucero, J. D. Gunn, V. L. Scarborough, Climate change and Classic Maya water management. *Water* **3**, 479–494 (2011).

55. S. Mardero, B. Schmook, J. O. López-Martinez, L. Cicero, C. Radel, Z. Christman, The uneven influence of climate trends and agricultural policies on maize production in the Yucatan Peninsula, Mexico. *Land* ***7****,* 80 (2018).

56. K. Carmean, N. Dunning, J. K. Kowalski, "High times in the Hill Country: The Terminal Classic Puuc region," in *The Terminal Classic in the Maya Lowlands: Collapse, Transition, and Transformation* A. Demarest, P. Rice, D. Rice, Eds. (University of Colorado Press, 2004), pp. 424–449.

57. R. Cobos, G. de Anda Alanís, R. G. Moll, Ancient climate and archaeology: Uxmal, Chichén Itzá, and their collapse at the end of the terminal classic period. *Archeol. Pap. Am. Anthropol. Assoc.* **24**, 56–71 (2014).

58. H. E. D. Pollock, *The Puuc: An Architectural Survey of the Hill Country of Yucatan and Northern Campeche, Mexico. Memoirs of the Peabody Museum, Harvard University Vol. 19.* (Harvard Univ. Press, 1980).

59. G. J. Bey, R. May Ciau, T. Gallareta Cervera, M. Galván Bernal, "Capítulo 3: Excavaciones Estratigraficas en los Grupos Yaxche y Kuche," in *Investigaciones Arqueológicas en las Ruinas de Kiuic y la Zona Labná-Kiuic, Distrito de Bolonchén, Yucatán, Mexico. Temporada de Campo 2010. Informe Técnico al Consejo de Arqueología del Instituto Nacional de Antropología e Historia, México* (INAH, 2010), pp. 3.1–3.65.

60. W. M. Ringle, T. Gallareta Negrón, R. May Ciau, K. E. Segilson, J. C. Fernandez-Diaz, D. Ortegón Zapata, Lidar survey of ancient Maya settlement in the Puuc region of Yucatán, Mexico. *PLOS ONE* **16**, e0249314 (2021).

61. S. R. Simms, E. Parker, G. J. Bey, T. Gallareta Negrón, Evidence from Escalera al Cielo: Abandonment of a Terminal Classic Puuc Maya Hill Complex in Yucatán, Mexico. *J. Field Archaeol.* **37**, 270–288 (2012)

62. G. E. Braswell, “The rise and fall of market exchange: A dynamic approach to ancient Maya economy,” in *Archaeological Approaches to Market Exchange in Ancient Societies*, C. P Garraty, B. L. Stark, Eds. (University Press of Colorado, 2010), pp. 127–140.

63. M. A. Masson, D. A. Freidel, An argument for Classic era Maya market exchange. *J. Anthropol. Archaeol.* **31**, 455–484 (2012).

64. R. Cobos, Chichen Itza and its economy at the end of the classic period: Tribute, centralized redistribution, and maritime stations. *Anc. Mesoam.* **34**, 1–23 (2023).

65. B. Volta, G. E. Braswell, “Alternative narratives and missing data: Refining the chronology of Chichen Itza,” in *The Maya and Their Central American Neighbors: Settlement Patterns, Architecture, Hieroglyphic Texts, and Ceramics*, G. E. Braswell, Ed. (Routledge, 2014), pp. 356–402.

66. W. M. Ringle, Debating Chichén Itzá. *Anc. Mesoam.* **28**, 119–136 (2017).

67. T. W. Stanton, K. A. Taube, J. D. Coltman, I. Marengo Camacho, Eds., *When East Meets West: Chichen Itza, Tula and the Postclassic Mesoamerican World. BAR International Series Vol.3134, Archaeology of the Maya Vol.12.* (British Archaeological Reports Publishing, 2023).

68. G. E. Braswell, N. Peniche May, “In the shadow to the pyramid: Excavations of the Great Platform of Chichen Itza,” in *The Ancient Maya of Mexico: Reinterpreting the Past of the Northern Maya Lowlands*, G. E. Braswell, Ed. (Equinox Publishing, 2012), pp. 229–263.

69. B. Volta, N. Peniche May, G. E. Braswell, “The archaeology of Chichen Itza: Its history, what we like to argue about, and what we think we know,” in *Landscapes of the Itza: Archaeology and Art History at Chichen Itza and Neighboring Sites*, L. Wren, C. Kristan-Graham, T. Nygard, K. Spencer, Eds. (University Press of Florida, 2018), pp. 28–64.

70. B. H. Dahlin, Climate change and the end of the Classic Period in Yucatan: Resolving a paradox. *Anc. Mesoam.* **13**, 327–340 (2002).

71. W. B. White, Speleothem microstructure/speleothem ontogeny: A review of Western contributions. *Int. J. Speleol.* **41**, 18 (2012).

72. C. C. Day, G. M. Henderson, Oxygen isotopes in calcite grown under cave-analogue conditions. *Geochim. Cosmochim. Acta* **75**, 3956–3972 (2011).

73. R. L. Edwards, J. H. Chen, G. J. Wasserburg, $^{238}$U-$^{234}$U-$^{230}$Th-$^{232}$Th systematics and the precise measurement of time over the past 500,000 years. *Earth Planet. Sci. Lett.* **81**, 175–192 (1987).

74. D. L. Hoffmann, $^{230}$Th isotope measurements of femtogram quantities for U-series dating using multi-ion counting (MIC) MC-ICPMS. *Int. J. Mass Spectrum.* **275**, 75–79 (2008).

75. A. Kaufman, W. Broecker, Comparison of Th$^{230}$ and C$^{14}$ ages for carbonate materials from Lakes Lahontan and Bonneville. *J. Geophys. Res.* **70**, 4039–4054 (1965).

76. D. A. Richards, J. A. Dorale, Uranium-series chronology and environmental applications of speleothems. *Rev. Mineral. Geochem.* **52**, 407–460 (2003).

77. H. Cheng, R. L. Edwards, C. C. Shen, V. J. Polyak, Y. Asmerom, J. Woodhead, J. Hellstrom, Y. Wang, X. Kong, C. Spötl, X. Wang, E. C. Alexander Jr., Improvements in $^{230}$Th dating, $^{230}$Th and $^{234}$U half-life values, and U–Th isotopic measurements by multi-collector inductively coupled plasma mass spectrometry. *Earth Planet. Sci. Lett.* **371**, 82–91 (2013).

78. J. Hellstrom, U–Th dating of speleothems with high initial 230Th using stratigraphical constraint. *Quat. Geochronol.* **1**, 289–295 (2006).

79. P. E. Carlson, N. R. Miller, J. L Banner, D. O. Breecker, R. C. Casteel, The potential of near-entrance stalagmites as high-resolution terrestrial paleoclimate proxies: Application of isotope and trace-element geochemistry to seasonally-resolved chronology. *Geochim Cosmochim. Acta* **235**, 55–75 (2018).

80. H. Hersbach, B. Bell, P. Berrisford, S. Hirahara, A. Horányi, J. Muñoz-Sabater, J. Nicolas, C. Peubey, R. Radu, D. Schepers, A. Simmons, C. Soci, S. Abdalla, X. Abellan, G. Balsamo, P. Bechtold, G. Biavati, J. Bidlot, M. Bonavita, G. De Chiara, P Dahlgren, D. Dee, M. Diamantakis,

R. Dragani, J. Flemming, R. Forbes, M. Fuentes, A. Geer, L. Haimberger, S. Healy, R. J. Hogan, E. Hólm, M. Janisková, S. Keeley, P. Laloyaux, P. Lopez, C. Lupu, G. Radnoti, P. de Rosnay, I. Rozum, F. Vamborg, S Villaume, J.-N. Thépaut, The ERA5 global reanalysis. *Q. J. Roy. Meteorol. Soc.* **146**, 1999–2049 (2020).

81. D. M. Tremaine, P. N. Froelich, Y. Wang, Speleothem calcite farmed in situ: Modern calibration of $\delta^{18}O$ and $\delta^{13}C$ paleoclimate proxies in a continuously monitored natural cave system. *Geochim. Cosmochim. Acta* **75**, 4929–4950 (2011).

82. Z. Sharp, *Principles of Stable Isotope Geochemistry* (Pearson Prentice Hall, 2007).

83. S. T. Kim, T. B. Coplen, J. Horita, Normalization of stable isotope data for carbonate minerals: Implementation of IUPAC guidelines. *Geochim. Cosmochim. Acta* **158**, 276–289 (2015).

84. H. P. Affek, S. Zaarur, Kinetic isotope effect in $CO_2$ degassing: Insight from clumped and oxygen isotopes in laboratory precipitation experiments. *Geochim. Cosmochim. Acta* **143**, 319–330 (2014).

85. M. Hansen, D. Scholz, B. R. Schöne, C. Spötl, Simulating speleothem growth in the laboratory: Determination of the stable isotope fractionation ($\delta^{13}C$ and $\delta^{18}O$) between $H_2O$, DIC and $CaCO_3$. *Chem. Geol.* **509**, 20–44 (2019).

86. M. P. Smyth, N. P. Dunning, E. M. Weaver, P. van Beynen, D. Ortegón Zapata, The perfect storm: Climate change and ancient Maya response in the Puuc Hills region of Yucatán. *Antiquity* **91**, 490–509 (2017).

87. N. P. Evans, T. K. Bauska, F. Gásquez-Sánchez, M. Brenner, J. H. Curtis, D. A. Hodell, Quantification of drought during the collapse of the Classic Maya civilization. *Science* **361**, 498–501 (2018).

88. J. Fohlmeister, N. R. G. Voarintsoa, F. A. Lechleitner, M. Boyd, S. Brandtstätter, M. J. Jacobson, J. L. Oster, Main controls on the stable carbon isotope composition of speleothems. *Geochim. Cosmochim. Acta* **279**, 67–87 (2020).

89. A. B. Frappier, D. Sahagian, L. A. González, S. J. Carpenter, El Niño events recorded by stalagmite carbon isotopes. *Science* **298**, 565–565 (2002).

90. A. B. Frappier, D. Sahagian, S. J. Carpenter, L. A. González, B. R. Frappier, Stalagmite stable isotope record of recent tropical cyclone events. *Geology* **35**, 111–114 (2007).

91. A. B. Frappier, Masking of interannual climate proxy signals by residual tropical cyclone rainwater: Evidence and challenges for low-latitude speleothem paleoclimatology. *Geochem. Geophys. Geosyst.* **14**, 3632–3647 (2013).

92. A. B. Frappier, J. Pyburn, A. D. Pinkey-Drobnis, X. Wang, D. Reide Corbett, B. H. Dahlin, Two millennia of tropical cyclone-induced mud layers in a northern Yucatán stalagmite: Multiple overlapping climatic hazards during the Maya Terminal Classic "megadroughts". *Geophys. Res. Lett.* **41**, 5148–5157 (2014).

93. C. M. Appendini, J. Hernández-Lasheras, R. Meza-Padilla, J. A. Kurczyn, Effect of climate change on wind waves generated by anticyclonic cold front intrusions in the Gulf of Mexico. *Climate Dynam.* **51**, 3747–3763 (2018).

94. J. A. Wassenburg, S. Riechelmann, A. Schröder-Ritzau, D. F. C. Riechelmann, D. K. Richter, A. Immenhauser, M. Terente, S. Constantin, A. Hachenberg, M. Hansen, D. Scholz, Calcite Mg and Sr partition coefficients in cave environments: Implications for interpreting prior calcite precipitation in speleothems. *Geochim. Cosmochim. Acta* **269**, 581–596 (2020).

95. Y. Huang, I. J. Fairchild, Partitioning of $Sr^{2+}$ and $Mg^{2+}$ into calcite under karst-analogue experimental conditions. *Geochim. Cosmochim. Acta* **65**, 47–62 (2001).

96. C. C. Day, G. M. Henderson, Controls on trace-element partitioning in cave-analogue calcite. *Geochim. Cosmochim. Acta* **120**, 612–627 (2013).

97. J. A. Wassenburg, D. Scholz, K. P. Jochum, H. Cheng, J. L. Oster, A. Immenhauser, D. K. Richter, T. Häger, R. A. Jamieson, J. U. L. Baldini, D. Hoffamn, S. F. M. Breitenbach, Determination of aragonite trace element distribution coefficients from speleothem calcite–aragonite transitions. *Geochim. Cosmochim. Acta* **190**, 347–367 (2016).

98. N. Scroxton, S. Burns, P. Dawson, J. M. Rhodes, K. Brent, D. McGee, H. Heijnis, P. Gadd, W. Hantoro, M. Gagan, Rapid measurement of strontium in speleothems using core-scanning micro-x-ray fluorescence. *Chem. Geol.* **487**, 12–22 (2019).

99. H. M. Stoll, C. C. Day, F. A. Lechleitner, O. Kost, L. Endres, J. Sliwinski, C. Pérez-Mejías, H. Cheng, D. Scholz, Distinguishing the combined vegetation and soil component of $\delta^{13}C$ variation in speleothem records from subsequent degassing and prior calcite precipitation effects. *Clim. Past* **19**, 2423–2444 (2023)

100. W. Dansgaard, Stable isotopes in precipitation. *Tellus* **16**, 436–468 (1964).
